# p73 regulates epidermal wound healing and induced keratinocyte programming

**DOI:** 10.1371/journal.pone.0218458

**Published:** 2019-06-19

**Authors:** J. Scott Beeler, Clayton B. Marshall, Paula I. Gonzalez-Ericsson, Timothy M. Shaver, Gabriela L. Santos Guasch, Spencer T. Lea, Kimberly N. Johnson, Hailing Jin, Bryan J. Venters, Melinda E. Sanders, Jennifer A. Pietenpol

**Affiliations:** 1 Department of Biochemistry, Vanderbilt University, Nashville, Tennessee, United States of America; 2 Vanderbilt-Ingram Cancer Center, Vanderbilt University Medical Center, Nashville, Tennessee, United States of America; 3 Department of Molecular Physiology and Biophysics, Vanderbilt University, Nashville, Tennessee, United States of America; 4 Department of Pathology, Microbiology and Immunology, Vanderbilt University Medical Center, Nashville, Tennessee, United States of America; Università degli Studi di Milano, ITALY

## Abstract

p63 is a transcriptional regulator of ectodermal development that is required for basal cell proliferation and stem cell maintenance. p73 is a closely related p53 family member that is expressed in select p63-positive basal cells and can heterodimerize with p63. p73-/- mice lack multiciliated cells and have reduced numbers of basal epithelial cells in select tissues; however, the role of p73 in basal epithelial cells is unknown. Herein, we show that p73-deficient mice exhibit delayed wound healing despite morphologically normal-appearing skin. The delay in wound healing is accompanied by decreased proliferation and increased levels of biomarkers of the DNA damage response in basal keratinocytes at the epidermal wound edge. In wild-type mice, this same cell population exhibited increased p73 expression after wounding. Analyzing single-cell transcriptomic data, we found that p73 was expressed by epidermal and hair follicle stem cells, cell types required for wound healing. Moreover, we discovered that p73 isoforms expressed in the skin (ΔNp73) enhance p63-mediated expression of keratinocyte genes during cellular reprogramming from a mesenchymal to basal keratinocyte-like cell. We identified a set of 44 genes directly or indirectly regulated by ΔNp73 that are involved in skin development, cell junctions, cornification, proliferation, and wound healing. Our results establish a role for p73 in cutaneous wound healing through regulation of basal keratinocyte function.

## Introduction

The p53 family of transcription factors (p53, p63, and p73) play critical roles in cell cycle regulation, DNA damage response, and cellular differentiation [1–9]. All three family members share structural and functional homology in their transactivation (TA), DNA binding, and oligomerization domains [10]. Due to the high degree of sequence homology in their DNA binding domains, family members bind to similar genomic regions and regulate overlapping target genes. Both p73 and p63 have two distinct promoters that encode for either a longer (TA) or shorter (ΔN) transactivation domain [7,9]. In general, TA isoforms induce canonical p53 activity (e.g. cell cycle arrest, DNA repair, and apoptosis) while ΔN isoforms can repress these activities by acting in a dominant-negative manner towards TA isoforms [9,11]. ΔN isoforms can also induce the expression of specific target genes on their own [12–14]. Adding further complexity, both TA and ΔN isoforms can be alternatively spliced in their C-terminus to produce variants (e.g. α, β, γ) with differing transcriptional activity [9,15]. In addition to sharing overlapping target genes, p73 and p63 can form stable heterodimers through association of their oligomerization domains [16,17].

The complicated interplay between p73 and p63 has made studying the individual roles of each protein challenging. The development of knockout mouse models has provided insight to the distinct biological roles of p63 and p73. Mice lacking p63 (p63-/-) fail to develop stratified epithelia, epithelial appendages, and limbs; and die shortly after birth due to desiccation [1,2]. Studies have collectively shown that p63 (specifically ΔNp63α) is expressed in basal progenitor cell populations of ectodermal tissues and is essential for stem cell maintenance, proliferation, and development [1,2,18,19]. Mice lacking p73 (p73-/-) have a diverse set of phenotypes including hydrocephalus, hippocampal dysgenesis, sterility, chronic infections, and premature aging [3,4,20–22]. Many of these phenotypes are primarily due to the loss of multiciliated cells, which require a TAp73-mediated transcriptional program to develop. We previously reported that 50% of p63-expressing basal epithelial cells in the trachea co-express p73 and that tracheas from p73-/- mice exhibit a 35% reduction in the number of basal epithelial cells [3]. However, the role of p73 in basal progenitor cells is largely unknown. Studies of somatic cell reprogramming have provided clues by showing that p73 is required for effective generation of induced pluripotent stem cells (iPSCs) with Yamanaka factors [23,24]. Mouse embryonic fibroblasts lacking p73 have impaired mesenchymal-to-epithelial transition (a rate-limiting step during reprogramming), resulting in iPSCs with a defective epithelial phenotype [23].

The skin has proven to be an excellent model system for studying basal cell function during homeostasis and after injury (e.g. wounding). The skin forms a barrier that protects the body from physical, microbial, and chemical assaults as well as unregulated loss of water and solutes [25]. The epidermis [also referred to herein as the interfollicular epidermis (IFE)] is the primary component of the skin barrier and consists of a multi-layered stratified epithelium with appendages. Basal stem cells in the innermost layer of the epidermis are essential for maintaining tissue homeostasis and give rise to cells that detach from the basal layer, exit the cell cycle, and undergo a differentiation program. During this process, keratinocytes migrate towards the surface of the skin and undergo a tightly controlled series of gene expression changes that result in the production of dead squames, which are essential for maintaining the skin barrier [26]. Following wounding, it is essential to quickly repair the epidermis and restore the barrier function of the skin. Stem cells of the skin, located in the basal layer of the epidermis and the hair follicle (HF) bulge, regulate this process [27]. Stem cells near the wound are activated to migrate into the wound bed and proliferate, helping reepithelialize the wound epidermis [28,29].

We used the skin as a model system to investigate the role of p73 in basal epithelial cells and discovered that p73 is required for timely healing of cutaneous wounds. Wounds in p73-/- mice healed slower and demonstrated decreased proliferation and increased levels of biomarkers associated with the DNA damage response in basal keratinocytes at the epidermal wound edge. In addition, p73 expression increased in the basal keratinocytes at the wound edge of p73+/+ mice. Through analysis of single-cell transcriptomic data, we found that p73 was expressed by epidermal and HF stem cells, the cell types that regulate wound healing. Using a model of somatic cell reprogramming, we determined that ΔNp73 enhances the expression of keratinocyte genes involved in skin development, proliferation, and wound healing.

## Results

### Analysis of p73 expression in human and murine skin

**Fig 1:**
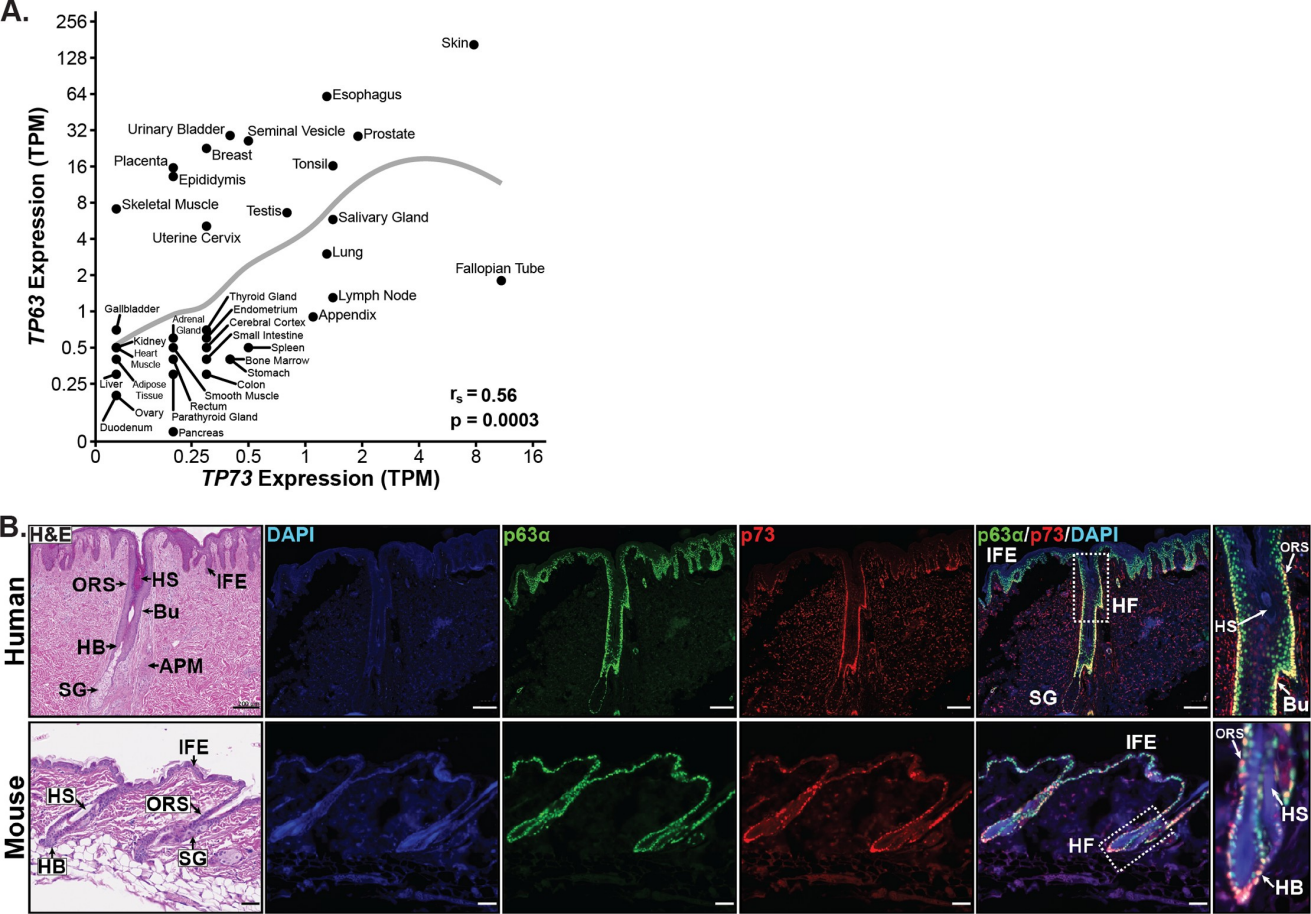
Analysis of p73 and p63 co-expression in human and murine skin. (A) Scatter plot of *TP63* versus *TP73* RNA-seq expression [units = transcripts per million (TPM)] by human tissue type (n = 37) from the Human Protein Atlas (172 total samples) [30]. Mean expression (TPM + 0.1) for each tissue is plotted on a log2 scale with a LOESS smooth local regression line (gray). Correlation between *TP63* and *TP73* was quantified using Spearman’s rank correlation coefficient (r_s_). (B) Representative micrographs of H&E and immunofluorescence (IF) staining on serial human (top) and mouse (bottom) skin sections; DAPI (blue), p63α (green), and p73 (red). Regions of the skin in micrographs are labeled as: interfollicular epidermis (IFE), hair follicle (HF), outer root sheath (ORS), HF bulge (Bu), hair bulb (HB), sebaceous gland (SG), hair shaft (HS), and arrector pili muscle (APM). Scale bars represent 200 μm for human and 50 μm for murine tissue. See also S1 and S2 Figs.

To determine which tissues express p73, we analyzed transcriptomic data from 37 human tissues [30]. We observed expression [>1 transcripts per million (TPM)] of *TP73* in many tissues with basal cell populations, including: skin, esophagus, prostate, tonsil, salivary gland, and lung (Fig 1A). Across all tissues, expression of *TP73* was significantly (p = 0.0003) correlated with expression of *TP63* (Fig 1A), a marker of basal epithelial cells. Given that *TP73* and *TP63* co-expression was highest in the skin and there are robust model systems for studying this organ, we focused our analysis on the role of p73 in the skin and its interplay with p63.

Prior work from our laboratory has shown that some pan-p73 antibodies cross-react with p63 [31]. In order to validate that the pan-p73 antibody (EP436Y) used in our studies did not cross-react with p63 and confound results, we conducted immunoblot analyses on a diverse set of primary and transformed human epithelial cells using p73 (EP436Y), p63 (AF1916), and p63α (H-129) antibodies (S1A Fig). Cells selected for analysis expressed varying levels of p73 and p63 in public databases and included a triple-negative breast cancer cell line that did not express either p73 or p63 mRNA (HCC70). p73 EP436Y recognized all four human p73 isoform controls, detected protein expression levels consistent with the known p73 and p63 mRNA levels, lacked non-specific detection in cells (HCC70) that do not express p73 RNA, and did not cross-react with p63 (S1A Fig). These results validate the specificity of p73 EP436Y and are consistent with previous reports of the antibody’s specificity in immunofluorescence (IF) analyses [3,21].

We performed IF staining for p73 and p63α on human and murine skin to determine cell expression patterns (Fig 1B). In both species, p73 expression was nuclear and limited to a subset of p63-positive cells in the basal layer of the IFE, outer root sheath (ORS) of the HF, hair bulb, and sebaceous gland (Fig 1B). We did not detect p73 expression in the hair shaft (HS) or the suprabasal layers of the IFE (Fig 1B). In human skin, p73 was strongly co-expressed with p63α in the stem cell compartment of the HF, termed the bulge (Fig 1B, top panel). The bulge is a specific area of the ORS containing HF stem cells that is located between the attachment site of the arrector pili muscle and the opening of the sebaceous gland [32]. We also saw expression of p73 in the bulge region of murine HFs. IF staining for p73 and p63α in murine skin (Fig 1B, bottom panel) was consistent with expression levels detected in the tissue by immunoblot analysis (S1B Fig). The IF results indicate that p73 expression in the skin is limited to a subset of p63-positive basal cells in the IFE and HF, and imply a role for p73 in basal keratinocytes.

### p73 is co-expressed with p63 throughout murine embryonic skin development

To determine if p73 is co-expressed with p63 during skin development in a pattern similar to that of adult murine tissue (Fig 1B, bottom panel), we performed IF staining for p73 and p63α in murine skin at several time points during embryogenesis. Similar to adult mice (Fig 1B, bottom panel), p73 was coordinately expressed with p63α in basal cells at all developmental time points analyzed (S1C Fig). During early embryonic stages (E12.5 and E13.5), p73 was expressed at low levels in the single-layered epidermis. At later time points during epidermal stratification (E14.5 and E16.5), expression of p73 increased and was restricted to cells in the basal layer, while p63α was expressed in both the basal and suprabasal layers (S1C Fig). HF morphogenesis is the process in which specific subsets of basal cells within the epidermis divide perpendicularly to the basement membrane and grow downward into the dermis to form HFs. p73 was highly expressed during HF morphogenesis (E16.5-P1) in the ORS and hair bulb of developing HFs (S1C Fig). By postnatal day 1 (P1), p73 expression in the basal IFE was reduced and limited to only a subset of cells (S1C Fig), similar to the IF staining seen in adult mice (Fig 1B, bottom panel).

### p73-/- murine skin has a normal morphological appearance

To evaluate if p73 plays a role in skin morphogenesis, we analyzed the skin of p73-/- mice [3,21]. Analysis of H&E-stained tissues showed no overt morphological differences between the back skin of adult p73+/+ and p73-/- mice (S2A Fig). Likewise, IF staining for markers of epidermal differentiation (K5, p63α, K14, E-cadherin, and K10) in p73+/+ and p73-/- mice demonstrated no significant difference in expression or localization (S2B–S2E Fig). HFs in S2 Fig that appear disconnected from the epidermis represent an artifact of sectioning. These results suggest that p73 is not required for proper epidermal morphogenesis or differentiation in mice.

### p73-/- mice exhibit delayed wound healing

**Fig 2:**
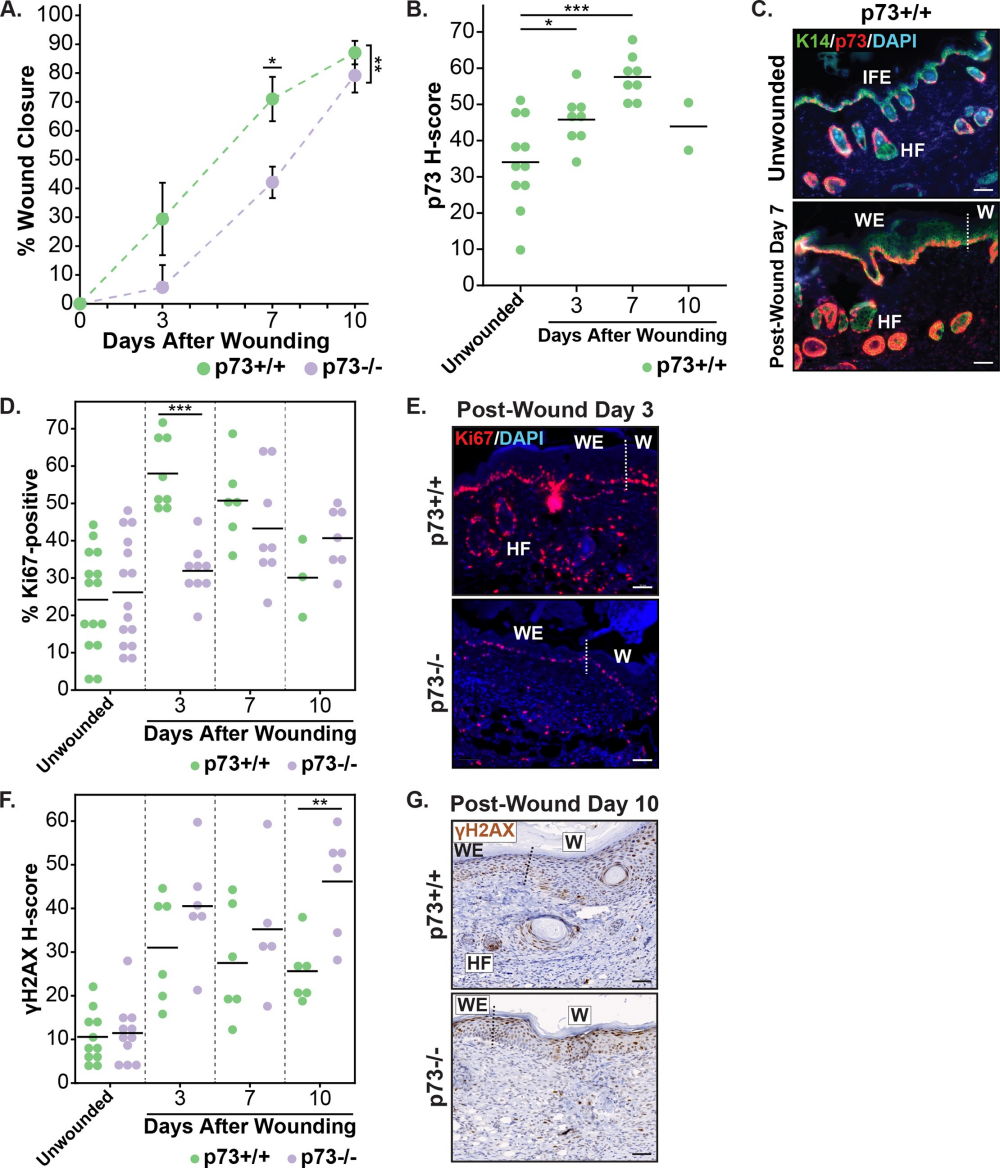
Biological and molecular analysis of cutaneous wound healing in p73+/+ and p73-/- mice. (A) Graph of percentage wound closure relative to initial wound size in p73+/+ and p73-/- mice at 0, 3, 7, and 10 days after wounding. The mean area of eight wounds is shown with error bars representing SEM. (B) Dot plot of p73 H-score in unwounded and wounded (days 3, 7, and 10) skin specimens from p73+/+ mice. (C) Representative micrographs of IF staining for K14 (green), p73 (red), and DAPI (blue) in unwounded and post-wound day 7 skin specimens from p73+/+ mice. (D) Dot plot of the percentage of Ki67-positive cells in unwounded and wounded (days 3, 7, and 10) skin specimens from p73+/+ and p73-/- mice. (E) Representative micrographs of IF staining for Ki67 (red) and DAPI (blue) in skin specimens from p73+/+ and p73-/- mice 3 days after wounding. (F) Dot plot of γH2AX H-score in unwounded and wounded (days 3, 7, and 10) skin specimens from p73+/+ and p73-/- mice. (G) Representative micrographs of immunohistochemistry (IHC) staining for γH2AX in skin specimens from p73+/+ and p73-/- mice 10 days after wounding. All scale bars represent 50 μm. In (B), (D), and (F), horizontal lines represent the mean. In (C), (E), and (G), regions of the skin are labeled as: IFE, HF, epidermal wound edge (WE), and newly-formed epidermis of the wound (W); and the dotted line indicates the border between the WE and W. *p-value < 0.05, **p-value < 0.01, ***p-value < 0.001. See also S3 and S4 Figs.

Given the lack of phenotypic differences in the skin of p73-/- mice under homeostatic conditions and the importance of basal keratinocytes, which express p73, in wound healing, we examined the role of p73 in the skin after epidermal wounding. We generated full-thickness wounds (diameter = 0.5 mm) on the backs of adult p73+/+ and p73-/- mice and analyzed the wound-healing process at post-wound days 0, 3, 7, and 10. Over the 10-day time course, the rate of wound closure was significantly (p = 0.004) decreased in p73-/- mice compared with p73+/+ mice, with the largest difference (p = 0.0125) occurring on post-wound day 7 (Fig 2A).

To gain insight to the molecular processes underlying the wound healing defect in p73-/- mice, we conducted IF and immunohistochemistry (IHC) staining on wounded skin sections from p73+/+ and p73-/- mice at post-wound days 3, 7, and 10. Semiquantitative scores of staining were generated by a pathologist using QuPath software [33] and reflect both the percentage of stained nuclei and staining intensity. In p73+/+ mice, p73 IF staining was increased in wounded compared to unwounded skin, and this difference was largest (p = 0.00007) at post-wound day 7 (Fig 2B). The increase of p73 expression after wounding was primarily due to increased expression in basal keratinocytes at the epidermal wound edge and in the newly-formed wound epidermis (Fig 2C and S3A Fig). These periwound basal keratinocytes had a diffuse and continuous p73 expression pattern, in contrast with the intermittent p73 expression detected in basal keratinocytes of unwounded skin (Fig 2C and S3A Fig). Adjacent normal (non-wounded) epidermis had less p73 IF staining than the epidermal wound edge (S3B Fig) and overall had a similar staining pattern as unwounded skin (Fig 2C, top panel).

Timely wound healing is dependent on a rapid proliferation response by basal keratinocytes at the epidermal wound edge. We found that expression of Ki67, a marker of proliferation, was increased in the skin after wounding by IF for both genotypes (Fig 2D). The increase of Ki67 expression in wounded skin was localized to the epidermal wound edge, adjacent HFs, and newly-formed wound epidermis (S4A Fig). At post-wound day 3, we detected a significant decrease (p = 0.00003) in the percentage of Ki67-positive cells in the skin of p73-/- compared to p73+/+ mice (Fig 2D and 2E), consistent with the observation that the largest difference in wound closure between the two genotypes occurred at post-wound day 7 (Fig 2A). The reduction of Ki67 staining in p73-/- wounds at post-wound day 3 was primarily due to decreased staining in the basal keratinocytes at the epidermal wound edge and in the newly-formed wound epidermis, and, to a lesser extent, adjacent HFs (Fig 2E). We did not detect a difference in Ki67 expression between the unwounded skin of p73+/+ and p73-/- mice (Figs 2D and S4A).

Previous work has shown that DNA damage contributes to the decline in stem cell function in aged tissues [34] and that p73 regulates the response to DNA damage [35–37]. γH2AX is a marker of the DNA damage response and replication stress [38,39]. In both p73+/+ and p73-/- mice, levels of γH2AX in the skin increased after wounding by IHC (Fig 2F and S4B Fig). The increase of γH2AX staining was detected in the epidermal wound edge, adjacent HFs, and newly-formed wound epidermis (S4B Fig). We observed a significant increase (p = 0.007) of γH2AX levels in p73-/- versus p73+/+ wounds at post-wound day 10 (Fig 2F and 2G). Collectively, these data establish a role for p73 in epidermal wound healing, in part through regulation of proliferation and the DNA damage response in basal keratinocytes.

### p73 is expressed by epidermal and HF stem cells

**Fig 3:**
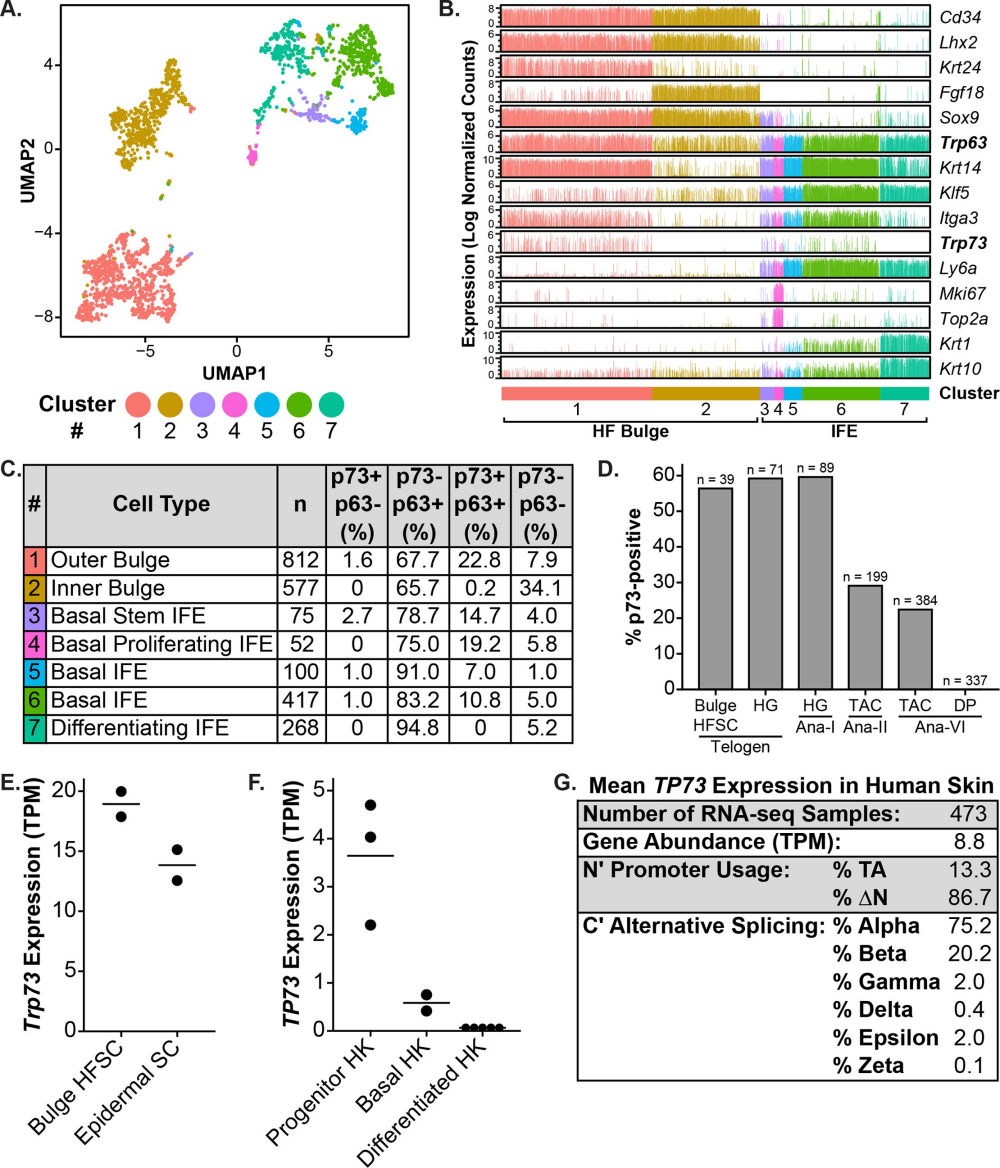
Analysis of p73 expression in epidermal and HF stem cells using transcriptomic data sets. (A) UMAP (Uniform Manifold Approximation and Projection) plot of 2310 murine keratinocyte (back skin) single cell transcriptomes from the Tabula Muris dataset [40]. Each dot represents an individual cell and is colored according to cluster membership. (B) Bar graphs of RNA expression for keratinocyte cell type markers from (A). Each bar represents one cell and is colored by cluster number from (A). (C) Table summarizing *Trp73* and *Trp63* expression and co-expression in single murine keratinocyte cells from (A) by cluster number. Each cluster number is annotated with: the primary cell type of the cluster (based on cell type marker expression), the number (n) of cells belonging to the cluster, and the percentage of cells with each p73/p63 expression status. (D) Bar graph of the percentage of p73-positive cells in different classes of murine HF cells by single-cell RNA-seq (scRNA-seq) [44]. Hair cycle stages include: telogen, anagen I (Ana-I), anagen II (Ana-II), and anagen VI (Ana-VI). Cell types include: bulge HF stem cells (HFSC), hair germ (HG) cells, transit-amplifying cells (TAC), and dermal papilla (DP) cells. The number of cells analyzed for each cell type is listed above each bar. (E) Dot plot of *Trp73* expression (TPM) in murine bulge HFSC and epidermal stem cells (SC) by bulk RNA-seq [45]. Cells were isolated by fluorescence-activated cell sorting (FACS) with marker-based sorting. Horizontal lines represent the mean. (F) Dot plot of *TP73* expression (TPM) in primary human keratinocytes (HK) grown and differentiated *in vitro* for 6 or 7 days [46,47]. HKs were enriched for progenitors based on rapid collagen IV adherence. Horizontal lines represent the mean. (G) Table with the mean *TP73* isoform expression in human skin (lower leg) RNA-seq samples (n = 473) from the Genotype-Tissue Expression (GTEx) Project [48,49]. For each sample, total gene abundance (TPM), N-terminal promoter usage, and C-terminal alternative splicing was calculated and is shown. See also S1 Table.

Epidermal and HF stem cells regulate tissue homeostasis and wound healing in the skin [27]. After epidermal injury, both epidermal and HF stem cells contribute to the wound-healing process by undergoing local migration and proliferation to ensure rapid repair of the epidermis and reestablishment of the skin barrier [28]. Based on the observation that the expression pattern of p73 overlaps with regions of the skin where the stem cell populations reside (Fig 1B) and that p73-/- mice have delayed epidermal wound healing (Fig 2A) due in part to decreased basal keratinocyte proliferation (Fig 2D), we determined p73 expression in epidermal and HF stem cells using transcriptomic data sets. We analyzed single-cell RNA-seq (scRNA-seq) data from 2310 murine back skin cells [40] that were isolated by *Itga6* (skin epithelial integrin) and/or *Cd34* (bulge HF stem cell marker) expression using fluorescence-activated cell sorting (FACS). Cluster analysis was performed with Seurat [41] and identified seven distinct cell clusters. We visualized the data using uniform manifold approximation and projection (UMAP) [42], a nonlinear dimensionality reduction technique that preserves both local and global data structure (Fig 3A). Cells were separated along UMAP1 based on their region of origin within the skin, either HF bulge (Cd34+) or IFE (Ly6a+; Fig 3A and 3B). HF bulge cells were divided into two clusters, one that expressed outer bulge markers (cluster #1, e.g. *Krt24*) and another that expressed inner bulge markers (cluster #2, e.g. *Fgf18*; Fig 3A and 3B) [43]. IFE cells were divided between five clusters (Fig 3A and 3B). Cluster #7 was distinguished by the expression of the differentiation markers *Krt1* and *Krt10* (Fig 3B). The remaining IFE clusters all expressed basal cell markers (e.g. *Krt14* and *Itga3*; Fig 3B). Among these, cluster #3 was characterized by stem markers (e.g. *Sox9*) and cluster #4 by proliferation markers (e.g. *Mki67* and *Top2a*; Fig 3B). Clusters #5 and #6 lacked distinguishing cell type markers (Fig 3B), but cluster #5 was unique in being the only cluster composed almost exclusively of cells from one stage (telogen) of the hair cycle (S1 Table).

We determined if each skin cell expressed *Trp73* and *Trp63* and summarized the results by cluster number (Fig 3C). Most cells (>85%) that expressed *Trp73* also expressed *Trp63* (Fig 3C). The percentage of cells with *Trp73* expression (p73+/p63- and p73+/p63+) was highest in the outer bulge cluster (Fig 3C), the stem cell compartment of the HF [32]. *Trp73* was expressed by a subset of cells in all of the basal IFE clusters (Fig 3C), the stem cell compartment of the epidermis. The basal proliferating IFE cluster had the second-highest percentage of cells that expressed *Trp73* (Fig 3C), consistent with a prior study that found increased expression of *Trp73* by non-label-retaining keratinocytes (i.e. those that divide more frequently) of the murine IFE [50]. The inner bulge and differentiating IFE clusters did not express *Trp73* (Fig 3C). *Trp73* was expressed in a lower percentage of cells within each cluster than *Trp63* (Fig 3C), consistent with its lower expression levels in the tissue (Fig 1A). *Trp63* was expressed by greater than 90% of cells in each cluster (p73-/p63+ and p73+/p63+) except for the inner bulge (Fig 3C).

Given that the outer bulge cluster had the highest percentage of cells that expressed p73 and p63 (Fig 3C), we decided to further study *Trp73* expression in defined HF stem cell populations. To do so, we analyzed an additional scRNA-seq dataset [44] that profiled diverse types of murine HF stem cells at different stages of the hair cycle. In telogen (quiescent stage), a large percentage of bulge HF stem cells and hair germ cells (56.4% and 59.2% respectively) expressed *Trp73* (Fig 3D). During the transition from telogen to anagen I, hair germ cells are activated to proliferate [51]; a large percentage of these cells (59.6%) retained expression of *Trp73* (Fig 3D). As anagen proceeds, the hair germ gives rise to transit-amplifying cells, which are highly proliferative and go on to produce the hair shaft and inner root sheath [52]. *Trp73* expression was lower in transit-amplifying cells during anagen II and VI (29.1% and 22.4% respectively; Fig 3D). Anagen VI dermal papilla cells (mesenchymal) did not express *Trp73*, as anticipated (Fig 3D). These results indicate that *Trp73* is expressed in HF stem cell populations during different stages of the hair cycle under homeostatic conditions, particularly bulge HF stem cells and hair germ cells.

scRNA-seq produces data with more technical noise and biological variation than bulk RNA-seq [53]. To validate the scRNA-seq results, we analyzed bulk RNA-seq data from murine bulge HF and epidermal stem cells isolated by FACS with marker-based sorting (*Itga6*, *Cd34*, and *Ly6a*) [45]. Both bulge HF and epidermal stem cells expressed *Trp73* (Fig 3E), consistent with the scRNA-seq results (Fig 3C and 3D). In order to assess *TP73* expression in human keratinocytes, we analyzed bulk RNA-seq data from primary human keratinocytes (HK) cultured and differentiated *in vitro* [46,47]. *TP73* was expressed by progenitor-enriched (isolated by rapid collagen IV adherence) and unenriched basal HKs, but not by HKs after 6 days of differentiation (Fig 3F), consistent with the p73 IF staining pattern observed in human skin (Fig 1B, top panel). These results collectively indicate that p73 is expressed by murine bulge HF and epidermal stem cells and basal HKs.

To gain insight to which p73 isoforms are expressed in the skin, we analyzed RNA-seq data from 473 human skin (lower leg) samples in the Genotype-Tissue Expression (GTEx) Project database [48,49]. Through analysis of exon-exon junction spanning reads, we found that ΔNp73 was more highly expressed than TAp73 (86.7% versus 13.3%), and that p73α and p73β were the predominantly expressed (75.2% and 20.2%, respectively) C-terminal splicing isoforms (Fig 3G). These results indicate that ΔNp73α and ΔNp73β are the predominantly expressed isoforms in the human skin during homeostasis.

### ΔNp73 enhances p63-mediated expression of keratinocyte genes during cellular reprogramming of human dermal fibroblasts to a basal keratinocyte-like state

Prior work has shown that p73 is required for effective generation of iPSCs with Yamanaka factors and that iPSCs lacking p73 have an attenuated epithelial phenotype [23]. To study the role of p73 in epidermal programming, we used an induced basal keratinocyte (iKC) model system first described by Chen and colleagues [54]. In this system, skin lineage-specific transcription factors KLF4 and ΔNp63α are expressed in neonatal human dermal fibroblasts (HDFn) to generate iKCs. To recapitulate the system, we infected HDFn cells with lentiviruses encoding KLF4, ΔNp63α, KLF4 + ΔNp63α, or empty vector controls (S5A Fig). We grew the cells for 3 days after the initial infection and performed RNA-seq. Cells infected with KLF4 + ΔNp63α lentivirus had increased expression of basal keratinocyte genes (e.g. *KRT14*, *ITGA3*) and reduced expression of fibroblast genes (e.g. *MME*, *VIM*) compared to control infections (S5B Fig and S2 Table). Also, the differentially expressed genes (n = 755) between HDFn cells infected with KLF4 + ΔNp63α versus control lentivirus were enriched for several Genome Ontology (GO) categories related to basal keratinocytes, including cell-substrate junction, extracellular structure organization, epidermis development, and epithelial cell development (S5C Fig and S3 Table). Our iKC results were consistent with those described in the original iKC report [54], indicating that we had a reliable model system in which to evaluate the role of ΔNp73 in induced basal keratinocyte programming.

**Fig 4:**
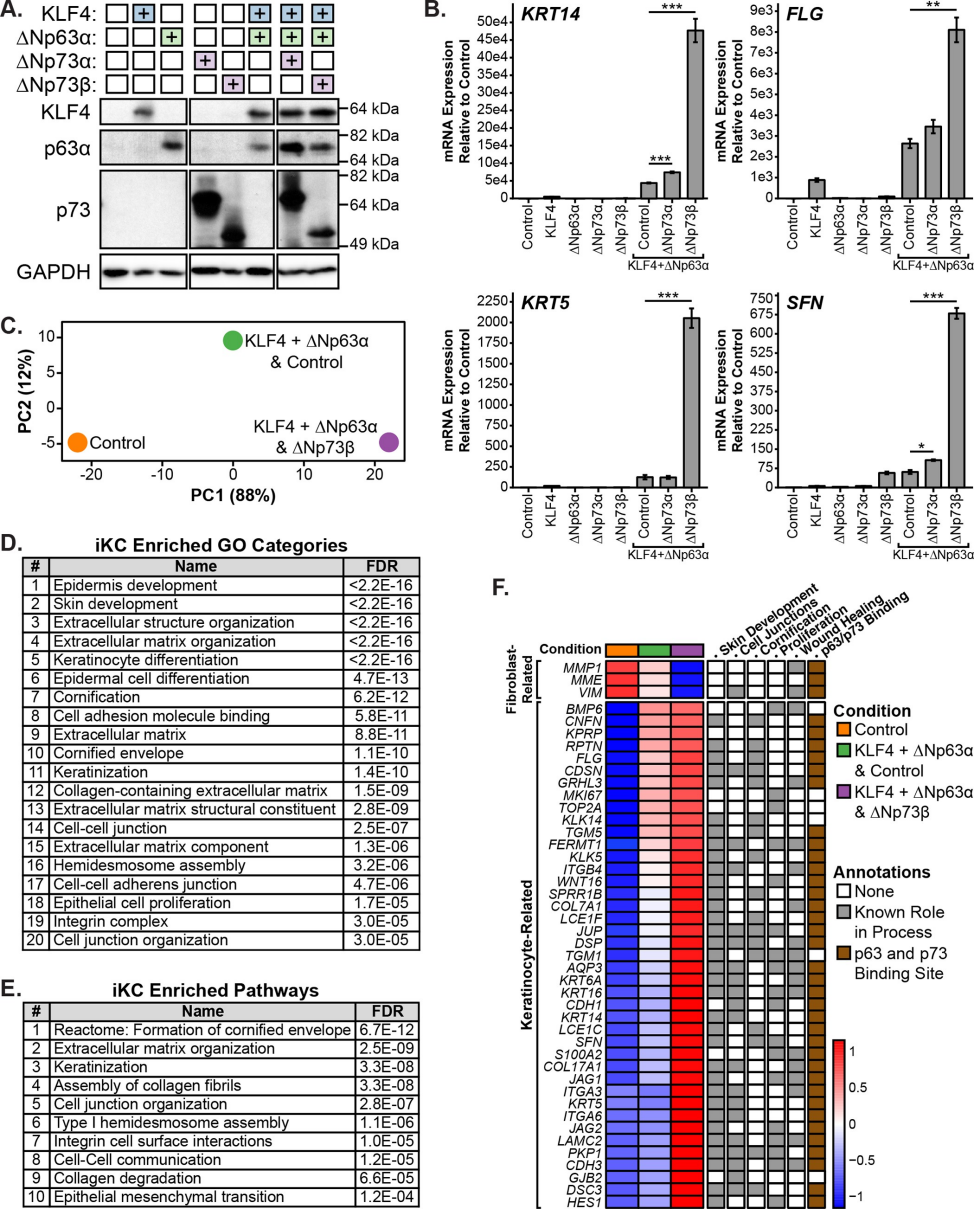
Analysis of ΔNp73 in epidermal programming using an induced basal keratinocyte (iKC) model system. (A) Immunoblot of KLF4, p63α, and p73 protein expression in neonatal human dermal fibroblast (HDFn) cells infected with lentivirus encoding ΔNp73 isoforms (ΔNp73α and ΔNp73β) or empty vector control in combination with KLF4 and ΔNp63α. Cells were grown for 3 days and protein was harvested for immunoblot analysis. (B) Bar graphs of RNA expression for the indicated keratinocyte genes in HDFn cells infected in (A). Cells were grown for 3 days and RNA was harvested for qRT-PCR analysis. Expression data are represented as the fold increase relative to control. The mean of three replicates is shown with error bars representing SEM. *p-value < 0.05, **p-value < 0.01, ***p-value < 0.001. (C) Principal component analysis (PCA) plot of RNA-seq from HDFn cells infected with lentivirus encoding ΔNp73β or empty vector control in combination with KLF4 and ΔNp63α. Cells were grown for 6 days and RNA was harvested for RNA-seq analysis. The percentage of variance contributed by each PC is listed in parentheses. (D and E) Tables listing the enriched Genome Ontology (GO) categories and pathways among the top 250 genes contributing to PC1 from (C). (F) Heatmap with the expression of a set of 44 genes that underlie the enrichment of GO categories from (D). Genes are annotated based on known roles in iKC-related processes (gray box) and the presence of a p63/p73 ChIP-seq peak within 50 kb of its TSS in multiple basal cell types (brown box). See also S5 and S6 Figs and S2–S9 Tables.

We infected HDFn cells with lentivirus encoding ΔNp73 isoforms (ΔNp73α and ΔNp73β) or empty vector control in combination with KLF4 + ΔNp63α and performed immunoblot analysis to verify protein expression (Fig 4A). We evaluated ΔNp73 isoforms because they were the most highly expressed isoforms in human skin samples (Fig 3G) and have been shown to regulate the initiation phase (involving mesenchymal-to-epithelial transition) of reprogramming murine embryonic fibroblasts into iPSCs [23]. Three days after infection of the HDFn cells with ΔNp73-expressing lentivirus, we harvested cells, isolated RNA, and performed qRT-PCR for keratinocyte genes that are markers of the iKC state (*KRT14*, *KRT5*, *SFN*, and *FLG*; Fig 4B). Co-expression of ΔNp73 isoforms with KLF4 + ΔNp63α led to increased expression of keratinocyte genes compared to KLF4 + ΔNp63α with empty vector control infections (Fig 4B). These results imply that ΔNp73 regulates the expression of keratinocyte genes in coordination with ΔNp63α and, in turn, conversion to the iKC state. For all four genes analyzed by qRT-PCR, we found that ΔNp73β induced higher levels of gene expression than ΔNp73α (Fig 4B). These results are consistent with published reports of ΔNp73β having greater transcriptional activity than ΔNp73α [14] and suggest that the role of ΔNp73 isoforms in the iKC model is due to transactivation of target gene expression.

In order to identify additional ΔNp73-regulated genes in the iKC model system, we infected HDFn cells with lentivirus encoding ΔNp73β isoforms or empty vector control in combination with KLF4 + ΔNp63α. We used ΔNp73β because it was the strongest inducer of keratinocyte gene expression in our qRT-PCR experiments (Fig 4B). After 6 days, we isolated RNA from the cells and performed RNA-seq. Consistent with our qRT-PCR analysis (Fig 4B), infection of HDFn cells with ΔNp73β in combination with KLF4 + ΔNp63α led to increased expression of *KRT14*, *KRT5*, *FLG*, and *SFN* as compared to KLF4 + ΔNp63α with control or control alone (S4 Table). Also, combination infections with ΔNp73β led to increased expression of additional basal keratinocyte genes (e.g. *ITGA3*, *ITGB4*, *KRT6A*, *KRT16*, *COL7A1*, and *CDH1*) and decreased expression of fibroblast genes (e.g. *VIM*, *MME*, and *MMP1*; S4 Table). We performed principal component analysis (PCA) on the RNA-seq data and the samples appeared to be ordered by degree of conversion to the iKC state along PC1, which was responsible for the majority (88%) of the variance in the data (Fig 4C). To further investigate, we determined the top 250 genes contributing to PC1 (S4 Table) and plotted their expression in a heatmap (S5D Fig). We identified two main sets of PC1 genes (S5D Fig). The larger subset of genes increased in expression along PC1 and included many basal keratinocyte-related genes (S5D Fig and S4 Table). The smaller subset of genes decreased in expression along PC1 and included many fibroblast-related genes (S5D Fig and S4 Table). These data suggest that the top 250 genes contributing to PC1 largely recapitulate the core genes involved in reprogramming to the iKC state and that ΔNp73β enhances this reprogramming process.

To determine gene sets associated with reprogramming HDFn cells to the iKC state, we performed enrichment analysis on the top 250 PC1 genes using WebGestalt [55]. The top 20 enriched Genome Ontology (GO) categories included many related to keratinocytes, including epidermis development, skin development, extracellular structure organization, cornification, cell adhesion molecule binding, and cell-cell junction (Fig 4D and S5 Table). Also, the top ten enriched pathways in WebGestalt contained many related to keratinocytes, including formation of the cornified envelope, extracellular matrix organization, keratinization, and cell junction organization (Fig 4E and S5 Table). To investigate the genes underlying the enriched GO categories in Fig 4D, we determined which of the top 250 PC1 genes were annotated with GO categories related to skin development, cell junctions, cornification, and proliferation. We identified a set of 44 genes involved in these biological processes that underlie most of the the observed GO category enrichment. Of interest, we found that ΔNp73β increased the expression of 18 genes with known roles in cellular proliferation, including *MKI67* and *TOP2A*, which is consistent with our data showing that p73-/- wounds have reduced proliferation (Fig 2D) and that basal proliferating cells have the highest percentage of p73 expression among IFE clusters (Fig 3C). Given the observed delay in wound healing in p73-/- mice (Fig 2A), we assessed if any of the core set of 44 genes had known roles in wound healing. We found that 18 of these genes have roles in wound healing, including *ITGA3* [56], *ITGB4* [57,58], *KRT6A* [59], *KRT16* [60,61], *COL7A1* [62], *S100A2* [63], *AQP3* [64], *TGM1* [65], *FERMT1* [66,67], and *GRHL3* [68] (Fig 4F).

To determine if p73 binds near the transcriptional start site (TSS) of any of the 44 genes involved in reprogramming to the iKC state, we analyzed p73 and p63 binding in ChIP-seq datasets from three basal cell models: HK (primary human keratinocytes; [46]), HaCaT (immortalized keratinocyte; newly generated herein), and HCC1806 (tumor-derived basal breast epithelial cells; newly generated herein and [21]). As part of our analyses, we leveraged previous findings that endogenous p73 and p63 have similar genomic binding profiles when co-expressed [69] and that heterotetramers of p73 and p63 are more thermodynamically stable than either homotetramer [70]. We also validated the previous finding by Yang and colleagues [69] using our own HCC1806 p73 and p63 ChIP-seq data (S6 Fig). Genes displayed in Fig 4F have an overlapping p63/p73 ChIP-seq peak within 50 kb of the TSS in two out of three cell types analyzed and the overlapping peak contained a canonical p63/p73 DNA binding motif (S6–S9 Tables). From the set of 44 genes involved in the iKC state, we found that 38 met our criteria for having a p63/p73 binding site (Fig 4F). Of immediate interest were 13 of these keratinocyte-related genes with known roles in wound healing (Fig 4F). Collectively, these findings support the conclusion that ΔNp73, in coordination with ΔNp63, acts as a regulator of the iKC state through direct and indirect regulation of key genes involved in skin development, cell junctions, cornification, proliferation, and wound healing.

### ΔNp73 enhances p63-mediated expression of keratinocyte genes during iKC reprogramming of mesenchymal breast cancer cells

To rule out a cell-type specific phenotype and determine if ΔNp73 could increase the expression of iKC marker genes in non-primary cells, we infected MDA-MB-231 cells (mesenchymal triple-negative breast cancer cell line) with lentivirus encoding ΔNp73 (ΔNp73α and ΔNp73β) or empty vector control in combination with KLF4 + ΔNp63α (S5E Fig). After 4 days we harvested cells, isolated RNA, and performed qRT-PCR for keratinocyte genes (*KRT14*, *KRT5*, *FLG*, and *SFN*) that were regulated in a p73-dependent manner in HDFn cells. Expression of ΔNp73 isoforms in combination with KLF4 + ΔNp63α had a similar effect in MDA-MB-231 cells (S5F Fig) as it did in HDFn cells (Fig 4B). ΔNp73 isoforms increased the expression of *KRT14*, *KRT5*, *FLG*, and *SFN*; for all four genes of these genes, ΔNp73β induced higher levels of expression than ΔNp73α (S5F Fig). These results indicate that ΔNp73 isoforms can regulate the expression of iKC marker genes in coordination with ΔNp63α in cell types from different tissues of origin as well as both primary and transformed cell states.

## Discussion

We discovered that p73 is required for timely cutaneous wound healing in mice. p73-/- mice exhibited delayed wound healing, due in part to decreased proliferation and increased activation of the DNA damage response in basal keratinocytes at the epidermal wound edge. In wild-type mice, this same cell population exhibited increased p73 expression after wounding. Further, we found that p73 was expressed by epidermal and HF stem cells, which regulate wound healing [27]. Using a model system for reprogramming fibroblasts to keratinocyte-like cells, we found that ΔNp73, in conjunction with ΔNp63, regulates the expression of keratinocyte genes and conversion to a keratinocyte-like state. We identified a core set of 44 genes directly or indirectly regulated by ΔNp73 that are involved in skin development, cell junctions, cornification, proliferation, and wound healing.

Adult tissue stem cells have an important role in tissue maintenance and regeneration after injury [71]. The stem cell function of these long-lived cells decreases with age due to the accumulation of cellular damage and contributes to the declining functional capacities observed in aging tissues [71]. The delayed healing, reduced proliferation, and increased levels of the DNA damage response observed in the epidermal wounds of p73-/- mice implies that p73 regulates the activity of adult skin stem cells after injury. While we did not detect an overt difference in the morphology or the expression pattern of epidermal differentiation markers in the skin of adult p73-/- mice, we cannot rule out that p73 also regulates adult skin stem cells during homeostasis, which in turn could impact the functional abilities of these cells after injury. A role for p73 in the regulation of adult stem cells is consistent with previous studies showing that p73 regulates neural stem cell maintenance [72,73] and protects against aging through regulation of cellular metabolism and the oxidative stress response [22,74,75]. Additional studies utilizing alternate wounding (e.g. partial-thickness wounding) and genetic (e.g. p73 isoform-specific knockout mice) models will be needed to further explore the roles of p73 in adult skin stem cells. Questions still unanswered include: (1) What roles does p73 play in epidermal and HF stem cells after injury and how do they differ between the cell types?; (2) What is the relative contribution of p73 isoforms to the wound healing phenotypes observed in p73-/- mice?; and (3) Does p73 have any roles in stem cell maintenance in the skin, similar to p63 [1,76]? Based on the increased p73 staining at post-wound day 3 in the newly-formed wound epidermis, a time point point at which basal keratinocytes are migrating into the wound to close the gap, and recent work demonstrating that p73 regulates migration [21,77], we hypothesize that p73 also regulates the migration of keratinocytes during epithelialization.

Through analysis of single cell expression in the skin (IF and scRNA-seq), we observed that nearly all p73-positive basal keratinocytes co-expressed p63. Since p73 and p63 can hetero-oligomerize [16,70], it is difficult to study the function of p73 in these cells without also taking into account p63. Moving forward, it will be important to characterize p73 and p63 isoform expression across tissues and in individual cells to better understand the interplay between these family members. Our ability to study p73 isoform expression in the single cell analyses was limited by the low number of N-terminal exon-exon spanning reads in current scRNA-seq data sets and the lack of p73 isoform-specific antibodies. From our analysis of GTEx data, we determined that ΔNp73 is the most highly expressed N-terminal isoform (both α and β) in adult human skin during homeostasis. Further studies are needed to determine if p73 isoform expression patterns vary amongst cell types in the skin (e.g. epidermal versus HF stem cells) and if ΔNp73 is the predominantly expressed isoform in basal cells across tissue types. If the latter is confirmed, it is tempting to posit that p73 isoform switching might be involved in signaling tracheal basal cells (ΔNp73+) to differentiate into multiciliated cells (TAp73+) [3].

In our experiments reprogramming fibroblasts into keratinocyte-like cells, we discovered that ΔNp73 isoforms, in particular ΔNp73β, significantly enhanced the expression of keratinocyte genes (e.g. *KRT14*, *KRT5*) and conversion to a keratinocyte-like state. Rather than causing gene expression changes in unique genes like KLF4, ΔNp73 largely amplified the magnitude of ΔNp63-induced gene expression, suggesting that ΔNp73 and ΔNp63 have similar transcriptional activity in this context. We observed similar results in reprogramming MDA-MB-231 cells, suggesting that this phenotype is not cell-type specific. Similarly, while validating the iKC model, we found that KLF4 significantly enhanced the expression of genes that were differentially expressed in ΔNp63-only conditions. Going forward, it will be important to determine how interplay between KLF4, ΔNp63, and ΔNp73 affects target gene regulation, as all three transcription factors are co-expressed by basal keratinocytes, and how ΔNp73 has such large effects on target gene expression in the iKC model system. Among the top ΔNp73-regulated genes, we identified an enrichment for genes involved in proliferation and wound healing; this knowledge may be useful in studying the mechanisms underlying the wound healing defect in p73-/- mice. Additional studies will be required to determine which of the ΔNp73-regulated genes are novel direct target genes in basal keratinocytes, as our analysis was limited to identifying genes that contained overlapping p63/p73 genomic binding sites within 50 kb of the TSS in multiple basal cell types. In future studies, it will be important to determine the role of TAp73 isoforms in the iKC model system and the skin more broadly, since these isoforms are generally more transcriptionally active, expressed in human skin, and deleted along with ΔNp73 isoforms in our p73-/- mice with the wound healing defect. Our reprogramming studies are limited by their *in vitro* nature and reliance on ectopic overexpression of lineage-specific transcription factors; nevertheless, the results are of significant value since they provide insight to the interplay between p73 and p63 in driving a keratinocyte-like transcriptional program.

Ankyloblepharon-ectodermal defects-cleft lip/palate (AEC) syndrome, a rare ectodermal dysplasia caused by heterozygous mutations in the C-terminus of *TP63*, is distinguished from other human *TP63*-associated disorders by the occurrence of severe skin erosions, especially those of the scalp [78,79]. A recent study determined that this unique symptom is due to the increased propensity of AEC-mutant p63 protein to aggregate intracellularly [80]. Interestingly, the authors found that AEC-mutant p63 bound to and coaggregated with p73, likely inhibiting its transcriptional activity. The overlapping functions between ΔNp73 and ΔNp63 in our studies with the iKC model system provide a potential explanation for the severe skin phenotype observed in patients with AEC syndrome. Namely, the skin erosions are more severe in patients with AEC syndrome because they impaired function of both p63 and p73. Analogously, the lack of an overt phenotype in the skin of p73-/- mice during homeostasis might be due to the ability of p63, which is more highly expressed, to compensate for the loss of p73.

In summary, our study provides insight to the role of p73 in basal keratinocytes and the skin overall and highlights the importance of studying the functional interplay of p73 and p63. Our results provide a mechanism for the wound healing phenotype observed in p73-/- mice and build on observations from previous studies linking ΔNp73 to stem cell activity in the basal cells of the trachea [3], iPSCs [23,24], neural stem cells [72], and cancer stem cells [81]. We propose a model in which ΔNp73 is required for effective function of adult skin stem cells after stress, through coordinate regulation with ΔNp63 of a progenitor cell transcriptional program.

## Materials and methods

### Animal model

We used previously described p73+/+ and p73-/- mice [3] in a BALB/c congenic background [21] for all experiments using murine tissue sections. This study was carried out in strict accordance with the recommendations in the Guide for the Care and Use of Laboratory Animals of the National Institutes of Health (NIH). The protocol was approved by the Institutional Animal Care and Use Committee (IACUC) of Vanderbilt University Medical Center. All surgery was performed under controlled-dose isoflurane anesthesia, and all efforts were made to minimize suffering. Mice were euthanized at the end of the study by isoflurane overdose followed by cervical dislocation.

### Immunofluorescence and immunohistochemistry

Immunostaining of tissue sections was performed as previously described [3]. Murine skin tissues were fixed in 10% neutral buffered formalin (NBF) and embedded in paraffin for sectioning. De-identified human skin sections were obtained from pre-existing de-identified formalin-fixed paraffin-embedded tissue blocks. These blocks were prepared from excess tissue remaining after evaluation and diagnosis at the time of a surgical procedure. The Vanderbilt University Medical Center Institutional Review Board considers these tissues exempt since they were pre-existing and de-identified. IF was conducted using the following antibodies: p73 EP436Y (Abcam, ab40658, 1:1000), p63α D2K8X (Cell Signaling Technology, 13109, 1:1000), Keratin 5 (Fitzgerald Industries International, 20R-CP003, 1:200), Keratin 14 (Fitzgerald Industries International, 20R-CP002, 1:200), E-cadherin (BD Biosciences, 610181, 1:1000), Keratin 10 (Abcam, ab76318, 1:100), and Ki67 B56 (BD Biosciences, 550609, 1:1000). p73, p63, and Ki67 were detected using TSA Plus Fluorescence Amplification Kit (PerkinElmer). Keratins (5, 10, 14) and E-cadherin were detected using species-appropriate Alexa Fluor secondary antibodies at 1:200 (Thermo Fisher Scientific). IHC was conducted using γH2AX (Novus Biologicals, NB100-2280, 1:1000) antibody.

### Cutaneous wounding assay

Mice (8–12 months old; ~1:1 ratio of males and females) were anesthetized using a controlled dose of isoflurane and four full-thickness wounds were made in the back skin using a 0.5 cm biopsy punch following Vanderbilt IACUC-approved protocols. Mice were monitored daily after wounding. Samples were collected at 0 (immediately after wounding), 3, 7, and 10 days after wounding with a total of eight wounds from two mice collected per time point. During sample collection, wounds were measured with calipers [wound area = (length/2) x (width/2) x π] and the back skin was harvested, fixed overnight in 10% NBF, and paraffin-embedded for further analysis. Whole tissue sections were digitally acquired using an AxioScan Z1 slide scanner (Carl Zeiss Canada). Automated semiquantitative scoring of staining in wounded and unwounded skin was performed on whole slide images by a pathologist who had not been informed of the study hypothesis using QuPath software [33]. Wound and unwounded areas were manually annotated. At least two areas for each condition (with a minimum total area of 1.94 mm^2^) were analyzed with the quantification algorithm to produce a semiquantitative score based on the percentage of stained nuclei (Ki67 IF) or the percentage of stained nuclei and staining intensity (p73 IF and γH2AX IHC). For the latter qualifications, H-scores were calculated using the following equation: H-score = 3*(% of 3+ intensity cells) + 2*(% of 2+ intensity cells) + 1*(% of 1+ intensity cells) [82]. Each selected region was visually assessed to verify correct performance of the quantification algorithm.

### Cell culture

CAL148 and MDA-MB-453 were grown in DMEM (Thermo Fisher Scientific, 11965–092) with 1 μg/mL EGF (Thermo Fisher Scientific, PHG0313) and 10% FBS (Gemini Bio Products, 100–106). HCC1806 (ATCC, CRL-2335) and HCC70 (ATCC, CRL-2315) cells were grown in RPMI + GlutaMAX (Thermo Fisher Scientific, 61870–036) with 10% FBS (Gemini Bio Products, 100–106). MCF10 (ATCC, CRL-10317) and HMEC cells were grown as previously described [83]. HaCaT (Cell Line Services, 300493), HaCaT C2 (single cell clone derived from parental HaCaT), HaCaT p63-/- (lack p63α expression as a result of CRISPR-Cas9 genomic editing of HaCaT C2 cells), 293FT (Thermo Fisher Scientific, R70007), and MDA-MB-231 (ATCC, HTB-26) cells were grown in DMEM (Thermo Fisher Scientific, 11965–092) with 10% FBS (Gemini Bio Products, 100–106). HDFn (ATCC, PCS-201-010) cells were grown in Medium 106 (Thermo Fisher Scientific, M-106-500) with 2% (v/v) Low Serum Growth Supplement (Thermo Fisher Scientific, S-003-10). All cell lines were grown in 100 U/mL Penicillin:Streptomycin (Gemini Bio Products, 400–109) and tested negative for *mycoplasma* (Lonza, LT07-418). HDFn cells were passaged using Trypsin-EDTA for Primary Cells (ATCC, PCS-999-003) and Trypsin Neutralizing Solution (ATCC, PCS-999-004). All other cells were passaged using 0.25% Trypsin-EDTA (Thermo Fisher Scientific, 25200–056).

### Cloning lentiviral expression vectors

The coding sequence (CDS) of KLF4, ΔNp63α, ΔNp73α, and ΔNp73β was amplified by PCR (while adding a restriction site to each end of the DNA) and cloned into pCDH-CMV-3xMCS-EF1-copGFP-T2A-puro by *Nhe*I (New England BioLabs, R3131S) and *Sal*I (New England BioLabs, R3138S) restriction digest and T7 DNA ligation (New England BioLabs, M0318S). Each expression vector was Sanger sequenced to ensure the CDS matched GENCODE annotations [84]. Further detail on all vectors including the source of the template DNA for each CDS is provided in S10 Table.

### Lentivirus production

Lentivirus was produced in 293FT cells through transfection of second-generation lentiviral vectors. Cells were plated in T-175 flasks and grown to 80–90% confluency.

For each lentivirus produced, the following vectors were diluted in 1.2 mL of Opti-MEM (Thermo Fisher Scientific, 31985–070): pMD2.G (1.68 pmol or 6.5 μg; Addgene, 12259), pxPAX2 (3.03 pmol or 21.4 μg; Addgene, 12260), and lentiviral transfer vector (3.83 pmol). Lipofectamine 2000 (140 μL; Thermo Fisher Scientific, 11668500) was diluted in 1.2 mL of Opti-MEM and incubated at room temperature for five minutes. Diluted DNA was added to the diluted Lipofectamine 2000 dropwise and incubated at room temperature for 30 minutes. Cells were switched to antibiotic-free media and the DNA-lipid complexes were added dropwise. The following morning, fresh media containing antibiotics was added. Virus was harvested 48 and 72 hours after transfection. Viral supernatant was centrifuged at 1000 rpm for 5 min, passed through a 0.45 μm syringe filter (Sarstedt, 83.1826), and stored at -80°C. Lentivirus was titered using the Lenti-X qRT-PCR Titration Kit (Takara Bio).

### Lentiviral infections

Lentivirus was stored at -80°C in aliquots prior to infection and was limited to one freeze-thaw cycle before use. Target cells were grown to 33–66% confluency in 6-well or 12-well plates before infection. For every infection condition, equally titered copy numbers of each virus were used in the presence of 6 μg/mL polybrene. Each plate was spun at 2,000 rpm for one hour at room temperature. The spinfection procedure was repeated 24 hours later and fresh media was added at 48 hours. Cells were grown for a total of 3–6 days after the initial infection before collection and the media was changed every other day.

### Immunoblotting

Cell pellets were collected by trypsinization and lysed in RIPA buffer supplemented with protease inhibitors (Sigma-Aldrich, 11697498001). Murine tissues were homogenized in RIPA buffer supplemented with protease inhibitors (Sigma-Aldrich, 11697498001) using a Diagenode Bioruptor with protein extraction beads (Diagenode, C20000021). The protein concentration of cultured cell and tissue extracts was quantified using the DC Protein Assay (Bio-Rad, 500–0116). Protein samples (30–50 μg for cells and 100–150 μg for tissues) were separated on 10% SDS-PAGE gels and transferred to polyvinylidene fluoride (PVDF) membranes (MilliporeSigma, IPVH00010). Membrane blocking and antibody incubations were conducted in 1X TBST with 5% w/v nonfat dry milk. Primary antibody incubations were performed overnight at 4°C with KLF4 (R&D Systems, AF3460, 1:2000), p63α H-129 (Santa Cruz Biotechnology, sc-8344, 1:500), p73 EP436Y (Abcam, ab40658, 1:1000), and GAPDH (Merck Millipore, MAB374, 1:10000). Secondary antibody incubations were performed the next day for 1 hour at room temperature using species-appropriate HRP-conjugated secondary antibodies (Thermo Fisher Scientific, 1:5000). Membranes were incubated in ECL substrate (Thermo Fisher Scientific, 32106) for 1–2 minutes and chemiluminescent signal was captured with X-ray film or Amersham Imager 600 (GE Healthcare Life Sciences, 29083461).

### RNA isolation

Total RNA was harvested from HDFn and MDA-MB-231 cell pellets that had been generated by cell trypsinization and frozen at -20°C. RNA for qRT-PCR analysis was isolated using the Aurum Total RNA Mini Kit (Bio-Rad, 732–6820). Samples were treated with DNase I for 30 minutes at room temperature. RNA for RNA-seq analysis was isolated using the RNAqueous-Micro Total RNA Isolation Kit (Thermo Fisher Scientific, AM1931). Samples were treated with DNase I for 20 minutes at room temperature. All RNA samples were quantified and assessed for quality using a NanoDrop One Spectrophotometer (Thermo Fisher Scientific, ND-ONE-W). Samples for RNA-seq were also analyzed using the Qubit RNA assay and BioAnalyzer 2100 or TapeStation system (Agilent).

### Quantitative reverse transcription PCR (qRT-PCR)

cDNA was generated from 750 ng of total RNA using MultiScribe Reverse Transcriptase (Thermo Fisher Scientific, 4311235) and oligo(dT) primer (Thermo Fisher Scientific, N8080128). Quantitative PCR was performed using the CXF96 Touch Real-Time PCR Detection System (BioRad, 1855195) with iQ SYBR Green Supermix (BioRad, 1708880). An annealing temperature of 60°C was used for each primer set. Samples were run in triplicate and normalized to *GAPDH*. The ΔΔCt method was used to calculate relative gene expression between samples [85]. Primer sequences for *KRT14*, *KRT5*, *FLG*, *SFN*, and *GAPDH* are listed in S10 Table.

### HDFn iKC RNA-seq

RNA-seq of HDFn iKC samples was performed at the Vanderbilt Technologies for Advanced Genomics (VANTAGE) core. Stranded RNA-seq libraries were prepared from total RNA using poly-A enrichment and the TruSeq RNA Library Preparation Kit (Illumina). Libraries were sequenced on an Illumina NovaSeq 6000 or NextSeq 500 using a paired-end 150 bp protocol. RNA-seq reads were trimmed to remove adapter sequences with Flexbar v3.4.0 [86] and aligned to hg19 (GRCh37 Primary Assembly) with STAR v2.6.1a [87] using default parameters and GENCODE v28lift37 annotations [84]. The number of reads mapped to GENCODE each gene was quantified with featureCounts v1.6.2 [88] and used with DESeq2 v1.14.1 [89] to perform differential expression analysis between samples. Overrepresentation enrichment analysis was conducted on differentially expressed genes (DESeq2 FDR < 0.1) using WebGestaltR v0.1.1 [55]. Transcript abundance (in TPM) was estimated using Kallisto v0.44.0 [90].

### HPA RNA-seq data analysis

RNA-seq transcript abundance data for 172 human samples across 37 tissue sites was downloaded from the Human Protein Atlas (HPA) on 8-3-2018 (v18) [30]. Transcript abundance had been estimated with Kallisto v0.42.4 [90] and gene-level abundance calculated as the sum of the TPM values for all protein-coding transcripts of a gene.

### Tabula Muris scRNA-seq data analysis

scRNA-seq count data from 2310 murine back skin cells was downloaded from the Tabula Muris Consortium [40] on 5-29-2018. RNA-seq libraries were prepared from individual FACS-sorted cells (Itga6+ and/or Cd34+) using the Smart-Seq2 protocol and sequenced on the Illumina NovaSeq 6000 (100 bp paired-end protocol). Sequencing reads had been aligned to the mm10plus genome using STAR v2.5.2b [87] and gene counts calculated with HTSEQ v0.6.1p1 [91]. scRNA-seq count data was analyzed with Seurat v2.3.4 [41]. Cells with less than 900 unique genes detected, less than 75000 total gene counts, or more than 12% ERCC RNA Control reads were excluded from the analysis. Expression data was log normalized and scaled to a mean of zero and variance of one for each gene. Cell-cell variation due to the total number of gene counts and percentage of ERCC reads was regressed out. PCA was performed with the top 2000 highly variable genes and the number of significant PCs for clustering analysis was determined using the JackStraw procedure and elbow plot analysis. Seurat identified a total of seven clusters using the first 33 principal components and a resolution of 0.4. Two-dimensional visualization of scRNA-seq data was performed with UMAP [42] using the first 33 principal components. UMAP parameters used included: n_neighbors = 15 and min_dist = 0.2. The expression of skin cell type markers (e.g. *Cd34*, *Ly6a*, *Fgf18*, *Krt14*, *Mki67*, *Krt10*) was analyzed for each cluster and used to assign cluster annotations. Cells were classified as expressing *Trp63* or *Trp73* if they had a log-normalized expression greater than or equal to 0.5.

### Hair follicle scRNA-seq data analysis

scRNA-seq gene expression data from 1119 murine hair follicle cells [44] were downloaded from the National Center for Biotechnology Information (NCBI) Gene Expression Omnibus database (GSE90848). Sequencing reads had been aligned to the mm10 using Bowtie2 v2.2.9 [92] and gene-level expression (TPM) quantified with RSEM [93]. Cells were classified as expressing *Trp63* or *Trp73* if they had expression greater than or equal to 1 TPM.

### Murine stem cell RNA-seq data analysis

RNA-seq data on murine bulge HF (GSM2656733 and GSM2656734) and epidermal (GSM2656735 and GSM2656736) stem cells isolated by FACS with marker-based sorting [45] were downloaded from the NCBI Sequence Read Archive (SRA) on 11-2-2018 (SRP093638). FASTQ files were processed as described in the HDFn iKC RNA-seq methods section except mm10 genome and GENCODE vM17 annotations [84] were used.

### Human keratinocyte RNA-seq data analysis

RNA-seq data on primary human keratinocytes grown and differentiated *in vitro* [46,47] were downloaded from the NCBI SRA on 10-24-2018 (SRP044925 and SRP070902). FASTQ files were processed as described in the HDFn iKC RNA-seq methods section. Differentiated samples used in the analysis were limited to those collected 6 or 7 days after the induction of differentiation.

### GTEx skin RNA-seq data analysis

RNA-seq data for 473 human lower leg skin samples were downloaded from the GTEx Portal on 1-9-2018 (V7 Release) [48,49]. Sequencing reads were aligned to hg19 with STAR v2.4.2a [87] using GENCODE v19 [94] annotations. Gene-level expression (units = TPM) had been quantified using RNA-SeQC v1.1.8 [95] and junction read counts using STAR v2.4.2a [87]. For each sample, counts of the reads spanning each *TP73* exon-exon junction were calculated and used to determine isoform expression. The percentage of N-terminal isoform expression was determined by the relative amounts of E3-E4 (TA) versus E3’-E4 (ΔN) junction counts. C-terminal *TP73* isoform expression was calculated in a similar manner. The percentage of p73α + β (E10-E11), p73γ + ε (E10-E12), p73ζ (E10-E13), and p73δ (E10-E14) was calculated by analyzing exon-exon junction counts starting at E10. The relative expression of p73α versus β was determined by comparing E12-E13 (α) and E12-E14 (β) junction counts.

### ChIP-seq

For each p63 ChIP, 25–50 million cells were crosslinked with formaldehyde (1–1.5%) for ten minutes at room temperature, collected by scraping, and sonicated to yield ~300 bp DNA fragments using a Diagenode Bioruptor. ChIP was performed with HaCaT and HCC1806 using 10 μg of p63α H-129 (Santa Cruz Biotechnology, sc-8344) antibody or with HaCaT C2 using 10 μg of p63 (AF1916, R&D Systems) antibody. Input control samples were collected from sonicated samples prior to performing ChIP. ChIP-seq libraries were prepared as described previously [3]. Illumina sequencing of ChIP-seq libraries was performed at the Vanderbilt VANTAGE core using a single-end 50 or 75 bp protocol. ChIP-seq reads were trimmed to remove adapter sequences using Flexbar v3.4.0 [86] and aligned to hg19 (GRCh37 Primary Assembly) using BWA v0.7.17-r1188 [96] with default parameters (BWA-backtrack for 50 bp reads and BWA-MEM for 75 bp reads). Duplicate reads were identified using the Picard v2.17.11 tool “MarkDuplicates”. Samtools v1.9 [97] was used to filter out duplicate, multimapping, improperly paired, and mitochondrial reads. Peak calling was performed with MACS2 [97,98] using input samples as a control and a FDR q-value threshold of 0.01. Motif enrichment analysis was performed with MEME-ChIP [99] on the genomic sequences at detected p63 peaks (length = 500 bp) as a QC step to ensure that the canonical p63/p73 DNA binding motif was identified and the ChIP was successful. Analysis of p73 and p63α genomic binding profiles in HCC1806 cells was conducted using deepTools [100]. p63/p73 binding sites within 50 kb of the TSS of the set of 44 genes involved in iKC reprogramming were determined by manually reviewing the MACS2-identified peaks in the Integrative Genomics Viewer (IGV) [101]. Genes were marked as containing a p63/p73 binding site (in Fig 4F) if an overlapping p63/p73 ChIP-seq peak was detected within 50 kb of the TSS in two out of three basal cell models analyzed (HK [46], HaCaT, and HCC1806 [21]) and the overlapping peak contained a canonical p63/p73 DNA binding motif.

### Statistical analysis

All statistical analyses and graphical representations were conducted using R (version 3.5.2 or 3.5.3) unless otherwise noted. The Spearman correlation between *TP63* and *TP73* expression across human tissues (HPA RNA-seq dataset) and its statistical significance was calculated using the "cor.test" function in R. Wound closure and qRT-PCR expression data are presented as the mean +/- SEM. The two-way ANOVA test was used to compute the mean difference in percentage wound closure between p73+/+ and p73-/- mice over days 0, 3, 7, and 10 (calculated with the R “aov” function using the genotype and days after wounding as factors). Student’s t-test was used to calculate statistical significance for wound closure on individual days, IF scores, IHC scores, and qRT-PCR expression (calculated with the R “t.test” function using the following parameters: two-sided, unpaired). Differences were considered significant when P < 0.05 and asterisks indicate: * = P < 0.05, ** = P < 0.01, and *** = P < 0.001.

## Supporting information

S1 Fig p73 and p63 protein expression in human epithelial cells, in murine tissues, and during murine embryonic skin development; related to Fig 1. (A) Immunoblot of p73 (EP436Y), p63 (AF1916), and p63α (H-129) protein expression in a diverse set of primary and transformed human epithelial cells with varying levels of p73 and p63 mRNA expression. Human p73 isoform controls (generated by ectopic overexpression in 293FT cells) were included in the analysis to assess the sensitivity and specificity of the pan-p73 antibody. HCC70 (triple-negative breast cancer) cells lack expression of p73 and p63 mRNA and were selected to evaluate antibody specificity. HaCaT p63α-/- cells lack p63α expression as a result of CRISPR-Cas9 genomic editing. (B) Immunoblot of p63α and p73 protein expression in murine skin and tongue. Tissue was harvested from p73+/+ mice for immunoblot analysis. (C) Representative micrographs of IF staining for DAPI (blue), p63α (green), and p73 (red) in skin specimens at the indicated stages of murine development. Scale bars represent 50 μm. Regions of the skin in micrographs are labeled as: epidermis (E), IFE, and HF.
(TIF)

S2 Fig Expression patterns of epidermal differentiation markers in p73+/+ and p73-/- murine skin, related to Fig 1. (A) Representative H&E micrographs of p73+/+ (top) and p73-/- (bottom) murine skin. (B-E) Representative micrographs of IF staining in p73+/+ (top) and p73-/- (bottom) murine skin for DAPI (blue) and: (B) K5 (green) and p63α (red), (C) K14 (green), (D) E-cadherin (red), (E) K10 (green). All scale bars represent 50 μm.
(TIF)

S3 Fig p73 IF analysis of p73+/+ skin after epidermal wounding, related to Fig 2. Representative micrographs of IF staining for DAPI (blue), K14 (green), and p73 (red) in skin specimens from p73+/+ mice: (A) unwounded and 3, 7, and 10 days after wounding, and (B) post-wound day 3 (zoomed out view). All scale bars represent 50 μm. Regions of the skin are labeled as: IFE, HF, epidermal wound edge (WE), newly-formed wound epidermis (W), and adjacent normal epidermis (AN). The white dotted lines indicate the border between the WE and W or AN.
(TIF)

S4 Fig Ki67 IF and γH2AX IHC analysis of p73+/+ and p73-/- skin after epidermal wounding, related to Fig 2. (A) Representative micrographs of IF staining for DAPI (blue) and Ki67 (red) in unwounded and wounded (days 3, 7, and 10) skin specimens from p73+/+ and p73-/- mice. (B) Representative micrographs of immunohistochemistry (IHC) staining for γH2AX in unwounded and wounded (days 3, 7, and 10) skin specimens from p73+/+ and p73-/- mice. All scale bars represent 50 μm. Regions of the skin are labeled as: IFE, HF, epidermal wound edge (WE), and newly-formed wound epidermis (W). The dotted lines indicate the border between the WE and W.
(TIF)

S5 Fig Validation of iKC model system and analysis of ΔNp73 in epidermal programming of mesenchymal breast cancer cells, related to Fig 4. (A) Immunoblot of KLF4 and p63α protein expression in HDFn cells infected with lentivirus encoding KLF4, ΔNp63α, KLF4 + ΔNp63α, or empty vector control. Cells were grown for 3 days and protein was harvested for immunoblot analysis. (B) Heatmap of the expression of iKC-related genes in HDFn cells infected with lentivirus encoding KLF4, ΔNp63α, KLF4 + ΔNp63α, or empty vector control. Cells were grown for 3 days and RNA was harvested for RNA-seq analysis. (C) Table listing the top 20 enriched GO categories for genes differentially expressed between KLF4 + ΔNp63α and empty vector control infections from (B). (D) Heatmap of the expression of the top 250 genes contributing to PC1 from Fig 4C. HDFn cells were infected with lentivirus encoding empty vector control, KLF4 + ΔNp63α & control, or KLF4 + ΔNp63α & ΔNp73β; grown for 6 days, and RNA was harvested for RNA-seq analysis. (E) Immunoblot of KLF4, p63α, and p73 protein expression in MDA-MB-231 cells infected with lentivirus encoding ΔNp73 isoforms (ΔNp73α and ΔNp73β) or empty vector control in combination with KLF4 and ΔNp63α. Cells were grown for 4 days and protein was harvested for immunoblot analysis. (F) Bar graphs of RNA expression for the indicated iKC marker genes in MDA-MB-231 cells infected in (E). Cells were grown for 4 days and RNA was harvested for qRT-PCR analysis. Expression data are represented as the fold increase relative to control. The mean of three replicates is shown with error bars representing SEM. *p-value < 0.05, **p-value < 0.01, ***p-value < 0.001.
(TIF)

S6 Fig Analysis of p73 and p63α genomic binding profiles in HCC1806 cells, related to Fig 4. Profile plots (top panel) and heatmaps (bottom panel) of p63α and p73 ChIP-seq signal in HCC1806 cells at p63α (two leftmost panels) and p73 (two rightmost panels) genomic binding sites. Correlation between p63α and p73 ChIP-seq signal was quantified using Pearson’s correlation coefficient (rp) with deepTools [100].
(TIF)

S1 Table Cluster analysis of murine back skin cells from Tabula Muris scRNA-seq dataset, related to Fig 3. (XLSX)

S2 Table Differential expression analysis between HDFn cells infected with control or KLF4 + ΔNp63α, related to S5 Fig. (XLSX)

S3 Table GO category enrichment analysis between HDFn cells infected with control or KLF4 + ΔNp63α, related to S5 Fig. (XLSX)

S4 Table PCA analysis of HDFn cells infected with control, KLF4 + ΔNp63α & control, or KLF4 + ΔNp63α & ΔNp73β; related to Fig 4. (XLSX)

S5 Table Gene set enrichment analysis of the top 250 iKC (PC1) genes from HDFn cells infected with control, KLF4 + ΔNp63α & control, or KLF4 + ΔNp63α & ΔNp73β; related to Fig 4. (XLSX)

S6 Table HaCaT p63 (AF1916) ChIP-seq genomic binding sites, related to Fig 4. (XLSX)

S7 Table HaCaT C2 p63α (H-129) ChIP-seq genomic binding sites, related to Fig 4. (XLSX)

S8 Table HCC1806 p63α (H-129) ChIP-seq genomic binding sites, related to Figs 4 and S6. (XLSX)

S9 Table HCC1806 p73 (EP436Y) ChIP-seq genomic binding sites, related to Figs 4 and S6. (XLSX)

S10 Table Key reagents. (XLSX)

S11 Table Supplemental table dictionary. (XLSX)

## References

[1] Yang A, Schweitzer R, Sun D, Kaghad M, Walker N, Bronson RT, et al p63 is essential for regenerative proliferation in limb, craniofacial and epithelial development. Nature. 1999;398: 714–718. 10.1038/19539 10227294

[2] Mills AA, Zheng B, Wang XJ, Vogel H, Roop DR, Bradley A. p63 is a p53 homologue required for limb and epidermal morphogenesis. Nature. 1999;398: 708–713. 10.1038/19531 10227293

[3] Marshall CB, Mays DJ, Beeler JS, Rosenbluth JM, Boyd KL, Santos Guasch GL, et al p73 Is Required for Multiciliogenesis and Regulates the Foxj1-Associated Gene Network. Cell Rep. 2016;14: 2289–2300. 10.1016/j.celrep.2016.02.035 26947080 PMC4794398

[4] Nemajerova A, Kramer D, Siller SS, Herr C, Shomroni O, Pena T, et al TAp73 is a central transcriptional regulator of airway multiciliogenesis. Genes Dev. 2016;30: 1300–1312. 10.1101/gad.279836.116 27257214 PMC4911929

[5] el-Deiry WS, Tokino T, Velculescu VE, Levy DB, Parsons R, Trent JM, et al WAF1, a potential mediator of p53 tumor suppression. Cell. 1993;75: 817–825. 8242752 10.1016/0092-8674(93)90500-p

[6] Westfall MD, Mays DJ, Sniezek JC, Pietenpol JA. The Delta Np63 alpha phosphoprotein binds the p21 and 14-3-3 sigma promoters in vivo and has transcriptional repressor activity that is reduced by Hay-Wells syndrome-derived mutations. Mol Cell Biol. 2003;23: 2264–2276. 10.1128/MCB.23.7.2264-2276.2003 12640112 PMC150720

[7] Kaghad M, Bonnet H, Yang A, Creancier L, Biscan JC, Valent A, et al Monoallelically expressed gene related to p53 at 1p36, a region frequently deleted in neuroblastoma and other human cancers. Cell. 1997;90: 809–819. 9288759 10.1016/s0092-8674(00)80540-1

[8] Osada M, Ohba M, Kawahara C, Ishioka C, Kanamaru R, Katoh I, et al Cloning and functional analysis of human p51, which structurally and functionally resembles p53. Nat Med. 1998;4: 839–843. 9662378 10.1038/nm0798-839

[9] Yang A, Kaghad M, Wang Y, Gillett E, Fleming MD, Dötsch V, et al p63, a p53 homolog at 3q27-29, encodes multiple products with transactivating, death-inducing, and dominant-negative activities. Mol Cell. 1998;2: 305–316. 9774969 10.1016/s1097-2765(00)80275-0

[10] Yang A, Kaghad M, Caput D, McKeon F. On the shoulders of giants: p63, p73 and the rise of p53. Trends Genet. 2002;18: 90–95. 11818141 10.1016/s0168-9525(02)02595-7

[11] Grob TJ, Novak U, Maisse C, Barcaroli D, Lüthi AU, Pirnia F, et al Human delta Np73 regulates a dominant negative feedback loop for TAp73 and p53. Cell Death Differ. 2001;8: 1213–1223. 10.1038/sj.cdd.4400962 11753569

[12] Ihrie RA, Marques MR, Nguyen BT, Horner JS, Papazoglu C, Bronson RT, et al Perp is a p63-regulated gene essential for epithelial integrity. Cell. 2005;120: 843–856. 10.1016/j.cell.2005.01.008 15797384

[13] Wu G, Nomoto S, Hoque MO, Dracheva T, Osada M, Lee C-CR, et al DeltaNp63alpha and TAp63alpha regulate transcription of genes with distinct biological functions in cancer and development. Cancer Res. 2003;63: 2351–2357. 12750249

[14] Liu G, Nozell S, Xiao H, Chen X. DeltaNp73beta is active in transactivation and growth suppression. Mol Cell Biol. 2004;24: 487–501. 10.1128/MCB.24.2.487-501.2004 14701724 PMC343790

[15] De Laurenzi V, Costanzo A, Barcaroli D, Terrinoni A, Falco M, Annicchiarico-Petruzzelli M, et al Two new p73 splice variants, gamma and delta, with different transcriptional activity. J Exp Med. 1998;188: 1763–1768. 10.1084/jem.188.9.1763 9802988 PMC2212516

[16] Davison TS, Vagner C, Kaghad M, Ayed A, Caput D, Arrowsmith CH. p73 and p63 are homotetramers capable of weak heterotypic interactions with each other but not with p53. J Biol Chem. 1999;274: 18709–18714. 10.1074/jbc.274.26.18709 10373484

[17] Joerger AC, Rajagopalan S, Natan E, Veprintsev DB, Robinson CV, Fersht AR. Structural evolution of p53, p63, and p73: implication for heterotetramer formation. Proc Natl Acad Sci U S A. 2009;106: 17705–17710. 10.1073/pnas.0905867106 19815500 PMC2764906

[18] Truong AB, Kretz M, Ridky TW, Kimmel R, Khavari PA. p63 regulates proliferation and differentiation of developmentally mature keratinocytes. Genes Dev. 2006;20: 3185–3197. 10.1101/gad.1463206 17114587 PMC1635152

[19] Senoo M, Pinto F, Crum CP, McKeon F. p63 Is essential for the proliferative potential of stem cells in stratified epithelia. Cell. 2007;129: 523–536. 10.1016/j.cell.2007.02.045 17482546

[20] Yang A, Walker N, Bronson R, Kaghad M, Oosterwegel M, Bonnin J, et al p73-deficient mice have neurological, pheromonal and inflammatory defects but lack spontaneous tumours. Nature. 2000;404: 99–103. 10.1038/35003607 10716451

[21] Santos Guasch GL, Beeler JS, Marshall CB, Shaver TM, Sheng Q, Johnson KN, et al p73 Is Required for Ovarian Follicle Development and Regulates a Gene Network Involved in Cell-to-Cell Adhesion. iScience. 2018;8: 236–249. 10.1016/j.isci.2018.09.018 30340069 PMC6197761

[22] Rufini A, Niklison-Chirou MV, Inoue S, Tomasini R, Harris IS, Marino A, et al TAp73 depletion accelerates aging through metabolic dysregulation. Genes Dev. 2012;26: 2009–2014. 10.1101/gad.197640.112 22987635 PMC3444727

[23] Martin-Lopez M, Maeso-Alonso L, Fuertes-Alvarez S, Balboa D, Rodríguez-Cortez V, Weltner J, et al p73 is required for appropriate BMP-induced mesenchymal-to-epithelial transition during somatic cell reprogramming. Cell Death Dis. 2017;8: e3034 10.1038/cddis.2017.432 28880267 PMC5636977

[24] Lin Y, Cheng Z, Yang Z, Zheng J, Lin T. DNp73 improves generation efficiency of human induced pluripotent stem cells. BMC Cell Biol. 2012;13: 9 10.1186/1471-2121-13-9 22449255 PMC3348002

[25] Proksch E, Brandner JM, Jensen J-M. The skin: an indispensable barrier. Exp Dermatol. 2008;17: 1063–1072. 19043850 10.1111/j.1600-0625.2008.00786.x

[26] Blanpain C, Fuchs E. Epidermal homeostasis: a balancing act of stem cells in the skin. Nat Rev Mol Cell Biol. 2009;10: 207–217. 10.1038/nrm2636 19209183 PMC2760218

[27] Ge Y, Fuchs E. Stretching the limits: from homeostasis to stem cell plasticity in wound healing and cancer. Nat Rev Genet. 2018;19: 311–325. 10.1038/nrg.2018.9 29479084 PMC6301069

[28] Ito M, Liu Y, Yang Z, Nguyen J, Liang F, Morris RJ, et al Stem cells in the hair follicle bulge contribute to wound repair but not to homeostasis of the epidermis. Nat Med. 2005;11: 1351–1354. 10.1038/nm1328 16288281

[29] Levy V, Lindon C, Zheng Y, Harfe BD, Morgan BA. Epidermal stem cells arise from the hair follicle after wounding. FASEB J. 2007;21: 1358–1366. 10.1096/fj.06-6926com 17255473

[30] Uhlen M, Oksvold P, Fagerberg L, Lundberg E, Jonasson K, Forsberg M, et al Towards a knowledge-based Human Protein Atlas. Nat Biotechnol. 2010;28: 1248–1250. 10.1038/nbt1210-1248 21139605

[31] Rosenbluth JM, Johnson K, Tang L, Triplett T, Pietenpol JA. Evaluation of p63 and p73 antibodies for cross-reactivity. Cell Cycle. 2009;8: 3702–3706. 10.4161/cc.8.22.10036 19855172

[32] Cotsarelis G, Sun TT, Lavker RM. Label-retaining cells reside in the bulge area of pilosebaceous unit: implications for follicular stem cells, hair cycle, and skin carcinogenesis. Cell. 1990;61: 1329–1337. 2364430 10.1016/0092-8674(90)90696-c

[33] Bankhead P, Loughrey MB, Fernández JA, Dombrowski Y, McArt DG, Dunne PD, et al QuPath: Open source software for digital pathology image analysis. Sci Rep. 2017;7: 16878 10.1038/s41598-017-17204-5 29203879 PMC5715110

[34] Oh J, Lee YD, Wagers AJ. Stem cell aging: mechanisms, regulators and therapeutic opportunities. Nat Med. 2014;20: 870–880. 10.1038/nm.3651 25100532 PMC4160113

[35] Wilhelm MT, Rufini A, Wetzel MK, Tsuchihara K, Inoue S, Tomasini R, et al Isoform-specific p73 knockout mice reveal a novel role for delta Np73 in the DNA damage response pathway. Genes Dev. 2010;24: 549–560. 10.1101/gad.1873910 20194434 PMC2841333

[36] Flores ER, Tsai KY, Crowley D, Sengupta S, Yang A, McKeon F, et al p63 and p73 are required for p53-dependent apoptosis in response to DNA damage. Nature. 2002;416: 560–564. 10.1038/416560a 11932750

[37] Tomasini R, Tsuchihara K, Wilhelm M, Fujitani M, Rufini A, Cheung CC, et al TAp73 knockout shows genomic instability with infertility and tumor suppressor functions. Genes Dev. 2008;22: 2677–2691. 10.1101/gad.1695308 18805989 PMC2559903

[38] Rogakou EP, Pilch DR, Orr AH, Ivanova VS, Bonner WM. DNA double-stranded breaks induce histone H2AX phosphorylation on serine 139. J Biol Chem. 1998;273: 5858–5868. 10.1074/jbc.273.10.5858 9488723

[39] Ward IM, Chen J. Histone H2AX is phosphorylated in an ATR-dependent manner in response to replicational stress. J Biol Chem. 2001;276: 47759–47762. 10.1074/jbc.C100569200 11673449

[40] Tabula Muris Consortium, Overall coordination, Logistical coordination, Organ collection and processing, Library preparation and sequencing, Computational data analysis, et al Single-cell transcriptomics of 20 mouse organs creates a Tabula Muris. Nature. 2018;562: 367–372. 10.1038/s41586-018-0590-4 30283141 PMC6642641

[41] Butler A, Hoffman P, Smibert P, Papalexi E, Satija R. Integrating single-cell transcriptomic data across different conditions, technologies, and species. Nat Biotechnol. 2018;36: 411–420. 10.1038/nbt.4096 29608179 PMC6700744

[42] Becht E, McInnes L, Healy J, Dutertre C-A, Kwok IWH, Ng LG, et al Dimensionality reduction for visualizing single-cell data using UMAP. Nat Biotechnol. 2018; 10.1038/nbt.4314 30531897

[43] Joost S, Zeisel A, Jacob T, Sun X, La Manno G, Lönnerberg P, et al Single-Cell Transcriptomics Reveals that Differentiation and Spatial Signatures Shape Epidermal and Hair Follicle Heterogeneity. Cell Syst. 2016;3: 221–237.e9. 10.1016/j.cels.2016.08.010 27641957 PMC5052454

[44] Yang H, Adam RC, Ge Y, Hua ZL, Fuchs E. Epithelial-Mesenchymal Micro-niches Govern Stem Cell Lineage Choices. Cell. 2017;169: 483–496.e13. 10.1016/j.cell.2017.03.038 28413068 PMC5510744

[45] Ge Y, Gomez NC, Adam RC, Nikolova M, Yang H, Verma A, et al Stem Cell Lineage Infidelity Drives Wound Repair and Cancer. Cell. 2017;169: 636–650.e14. 10.1016/j.cell.2017.03.042 28434617 PMC5510746

[46] Kouwenhoven EN, Oti M, Niehues H, van Heeringen SJ, Schalkwijk J, Stunnenberg HG, et al Transcription factor p63 bookmarks and regulates dynamic enhancers during epidermal differentiation. EMBO Rep. 2015;16: 863–878. 10.15252/embr.201439941 26034101 PMC4515125

[47] Cavazza A, Miccio A, Romano O, Petiti L, Malagoli Tagliazucchi G, Peano C, et al Dynamic Transcriptional and Epigenetic Regulation of Human Epidermal Keratinocyte Differentiation. Stem Cell Reports. 2016;6: 618–632. 10.1016/j.stemcr.2016.03.003 27050947 PMC4834057

[48] GTEx Consortium. The Genotype-Tissue Expression (GTEx) project. Nat Genet. 2013;45: 580–585. 10.1038/ng.2653 23715323 PMC4010069

[49] Carithers LJ, Ardlie K, Barcus M, Branton PA, Britton A, Buia SA, et al A Novel Approach to High-Quality Postmortem Tissue Procurement: The GTEx Project. Biopreserv Biobank. 2015;13: 311–319. 10.1089/bio.2015.0032 26484571 PMC4675181

[50] Sada A, Jacob F, Leung E, Wang S, White BS, Shalloway D, et al Defining the cellular lineage hierarchy in the interfollicular epidermis of adult skin. Nat Cell Biol. 2016;18: 619–631. 10.1038/ncb3359 27183471 PMC4884151

[51] Greco V, Chen T, Rendl M, Schober M, Pasolli HA, Stokes N, et al A two-step mechanism for stem cell activation during hair regeneration. Cell Stem Cell. 2009;4: 155–169. 10.1016/j.stem.2008.12.009 19200804 PMC2668200

[52] Hsu Y-C, Li L, Fuchs E. Transit-amplifying cells orchestrate stem cell activity and tissue regeneration. Cell. 2014;157: 935–949. 10.1016/j.cell.2014.02.057 24813615 PMC4041217

[53] Chen G, Ning B, Shi T. Single-Cell RNA-Seq Technologies and Related Computational Data Analysis. Front Genet. 2019;10: 47 10.3389/fgene.2019.00047 31024627 PMC6460256

[54] Chen Y, Mistry DS, Sen GL. Highly Rapid and Efficient Conversion of Human Fibroblasts to Keratinocyte-Like Cells. J Invest Dermatol. 2014;134: 335–344. 10.1038/jid.2013.327 23921950 PMC3875612

[55] Wang J, Vasaikar S, Shi Z, Greer M, Zhang B. WebGestalt 2017: a more comprehensive, powerful, flexible and interactive gene set enrichment analysis toolkit. Nucleic Acids Res. 2017;45: W130–W137. 10.1093/nar/gkx356 28472511 PMC5570149

[56] Reynolds LE, Conti FJ, Silva R, Robinson SD, Iyer V, Rudling R, et al alpha3beta1 integrin-controlled Smad7 regulates reepithelialization during wound healing in mice. J Clin Invest. 2008;118: 965–974. 10.1172/JCI33538 18246199 PMC2215730

[57] Liu C, Liu H-J, Xiang Y, Tan Y-R, Zhu X-L, Qin X-Q. Wound repair and anti-oxidative capacity is regulated by ITGB4 in airway epithelial cells. Mol Cell Biochem. 2010;341: 259–269. 10.1007/s11010-010-0457-y 20364299

[58] Turcan I, Pasmooij AMG, van den Akker PC, Lemmink H, Halmos GB, Sinke RJ, et al Heterozygosity for a Novel Missense Mutation in the ITGB4 Gene Associated With Autosomal Dominant Epidermolysis Bullosa. JAMA Dermatol. 2016;152: 558–562. 10.1001/jamadermatol.2015.5236 26817667

[59] Wojcik SM, Bundman DS, Roop DR. Delayed wound healing in keratin 6a knockout mice. Mol Cell Biol. 2000;20: 5248–5255. 10.1128/mcb.20.14.5248-5255.2000 10866680 PMC85973

[60] Patel GK, Wilson CH, Harding KG, Finlay AY, Bowden PE. Numerous keratinocyte subtypes involved in wound re-epithelialization. J Invest Dermatol. 2006;126: 497–502. 10.1038/sj.jid.5700101 16374449

[61] Lessard JC, Piña-Paz S, Rotty JD, Hickerson RP, Kaspar RL, Balmain A, et al Keratin 16 regulates innate immunity in response to epidermal barrier breach. Proc Natl Acad Sci U S A. 2013;110: 19537–19542. 10.1073/pnas.1309576110 24218583 PMC3845144

[62] Nyström A, Velati D, Mittapalli VR, Fritsch A, Kern JS, Bruckner-Tuderman L. Collagen VII plays a dual role in wound healing. J Clin Invest. 2013;123: 3498–3509. 10.1172/JCI68127 23867500 PMC3726167

[63] Pan S-C, Li C-Y, Kuo C-Y, Kuo Y-Z, Fang W-Y, Huang Y-H, et al The p53-S100A2 Positive Feedback Loop Negatively Regulates Epithelialization in Cutaneous Wound Healing. Sci Rep. 2018;8: 5458 10.1038/s41598-018-23697-5 29615682 PMC5882638

[64] Hara-Chikuma M, Verkman AS. Aquaporin-3 facilitates epidermal cell migration and proliferation during wound healing. J Mol Med. 2008;86: 221–231. 10.1007/s00109-007-0272-4 17968524

[65] Inada R, Matsuki M, Yamada K, Morishima Y, Shen SC, Kuramoto N, et al Facilitated wound healing by activation of the Transglutaminase 1 gene. Am J Pathol. 2000;157: 1875–1882. 10.1016/S0002-9440(10)64826-2 11106560 PMC1885758

[66] Jobard F, Bouadjar B, Caux F, Hadj-Rabia S, Has C, Matsuda F, et al Identification of mutations in a new gene encoding a FERM family protein with a pleckstrin homology domain in Kindler syndrome. Hum Mol Genet. 2003;12: 925–935. 10.1093/hmg/ddg097 12668616

[67] Siegel DH, Ashton GHS, Penagos HG, Lee JV, Feiler HS, Wilhelmsen KC, et al Loss of kindlin-1, a human homolog of the Caenorhabditis elegans actin-extracellular-matrix linker protein UNC-112, causes Kindler syndrome. Am J Hum Genet. 2003;73: 174–187. 10.1086/376609 12789646 PMC1180579

[68] Caddy J, Wilanowski T, Darido C, Dworkin S, Ting SB, Zhao Q, et al Epidermal wound repair is regulated by the planar cell polarity signaling pathway. Dev Cell. 2010;19: 138–147. 10.1016/j.devcel.2010.06.008 20643356 PMC2965174

[69] Yang A, Zhu Z, Kettenbach A, Kapranov P, McKeon F, Gingeras TR, et al Genome-wide mapping indicates that p73 and p63 co-occupy target sites and have similar dna-binding profiles in vivo. PLoS One. 2010;5: e11572 10.1371/journal.pone.0011572 20644729 PMC2904373

[70] Gebel J, Luh LM, Coutandin D, Osterburg C, Löhr F, Schäfer B, et al Mechanism of TAp73 inhibition by ΔNp63 and structural basis of p63/p73 hetero-tetramerization. Cell Death Differ. 2016;23: 1930–1940. 10.1038/cdd.2016.83 27716744 PMC5136491

[71] Ermolaeva M, Neri F, Ori A, Rudolph KL. Cellular and epigenetic drivers of stem cell ageing. Nat Rev Mol Cell Biol. 2018;19: 594–610. 10.1038/s41580-018-0020-3 29858605

[72] Talos F, Abraham A, Vaseva AV, Holembowski L, Tsirka SE, Scheel A, et al p73 is an essential regulator of neural stem cell maintenance in embryonal and adult CNS neurogenesis. Cell Death Differ. 2010;17: 1816–1829. 10.1038/cdd.2010.131 21076477 PMC3260880

[73] Agostini M, Tucci P, Chen H, Knight RA, Bano D, Nicotera P, et al p73 regulates maintenance of neural stem cell. Biochem Biophys Res Commun. 2010;403: 13–17. 10.1016/j.bbrc.2010.10.087 20977890 PMC3041924

[74] Du W, Jiang P, Mancuso A, Stonestrom A, Brewer MD, Minn AJ, et al TAp73 enhances the pentose phosphate pathway and supports cell proliferation. Nat Cell Biol. 2013;15: 991–1000. 10.1038/ncb2789 23811687 PMC3733810

[75] Marini A, Rotblat B, Sbarrato T, Niklison-Chirou MV, Knight JRP, Dudek K, et al TAp73 contributes to the oxidative stress response by regulating protein synthesis. Proc Natl Acad Sci U S A. 2018;115: 6219–6224. 10.1073/pnas.1718531115 29844156 PMC6004440

[76] Su X, Paris M, Gi YJ, Tsai KY, Cho MS, Lin Y-L, et al TAp63 prevents premature aging by promoting adult stem cell maintenance. Cell Stem Cell. 2009;5: 64–75. 10.1016/j.stem.2009.04.003 19570515 PMC3418222

[77] Fernandez-Alonso R, Martin-Lopez M, Gonzalez-Cano L, Garcia S, Castrillo F, Diez-Prieto I, et al p73 is required for endothelial cell differentiation, migration and the formation of vascular networks regulating VEGF and TGFβ signaling. Cell Death Differ. 2015;22: 1287–1299. 10.1038/cdd.2014.214 25571973 PMC4495354

[78] McGrath JA, Duijf PH, Doetsch V, Irvine AD, de Waal R, Vanmolkot KR, et al Hay-Wells syndrome is caused by heterozygous missense mutations in the SAM domain of p63. Hum Mol Genet. 2001;10: 221–229. 10.1093/hmg/10.3.221 11159940

[79] Julapalli MR, Scher RK, Sybert VP, Siegfried EC, Bree AF. Dermatologic findings of ankyloblepharon-ectodermal defects-cleft lip/palate (AEC) syndrome. Am J Med Genet A. 2009;149A: 1900–1906. 10.1002/ajmg.a.32797 19681128

[80] Russo C, Osterburg C, Sirico A, Antonini D, Ambrosio R, Würz JM, et al Protein aggregation of the p63 transcription factor underlies severe skin fragility in AEC syndrome. Proc Natl Acad Sci U S A. 2018;115: E906–E915. 10.1073/pnas.1713773115 29339502 PMC5798343

[81] Meier C, Hardtstock P, Joost S, Alla V, Pützer BM. p73 and IGF1R Regulate Emergence of Aggressive Cancer Stem-like Features via miR-885-5p Control. Cancer Res. 2016;76: 197–205. 10.1158/0008-5472.CAN-15-1228 26554827

[82] McCarty KS Jr, Miller LS, Cox EB, Konrath J, McCarty KS Sr. Estrogen receptor analyses. Correlation of biochemical and immunohistochemical methods using monoclonal antireceptor antibodies. Arch Pathol Lab Med. 1985;109: 716–721. 3893381

[83] Hearnes JM, Mays DJ, Schavolt KL, Tang L, Jiang X, Pietenpol JA. Chromatin immunoprecipitation-based screen to identify functional genomic binding sites for sequence-specific transactivators. Mol Cell Biol. 2005;25: 10148–10158. 10.1128/MCB.25.22.10148-10158.2005 16260627 PMC1280257

[84] Frankish A, Diekhans M, Ferreira A-M, Johnson R, Jungreis I, Loveland J, et al GENCODE reference annotation for the human and mouse genomes. Nucleic Acids Res. 2019;47: D766–D773. 10.1093/nar/gky955 30357393 PMC6323946

[85] Livak KJ, Schmittgen TD. Analysis of relative gene expression data using real-time quantitative PCR and the 2(-Delta Delta C(T)) Method. Methods. 2001;25: 402–408. 10.1006/meth.2001.1262 11846609

[86] Dodt M, Roehr JT, Ahmed R, Dieterich C. FLEXBAR-Flexible Barcode and Adapter Processing for Next-Generation Sequencing Platforms. Biology. 2012;1: 895–905. 10.3390/biology1030895 24832523 PMC4009805

[87] Dobin A, Davis CA, Schlesinger F, Drenkow J, Zaleski C, Jha S, et al STAR: ultrafast universal RNA-seq aligner. Bioinformatics. 2013;29: 15–21. 10.1093/bioinformatics/bts635 23104886 PMC3530905

[88] Liao Y, Smyth GK, Shi W. featureCounts: an efficient general purpose program for assigning sequence reads to genomic features. Bioinformatics. 2014;30: 923–930. 10.1093/bioinformatics/btt656 24227677

[89] Love MI, Huber W, Anders S. Moderated estimation of fold change and dispersion for RNA-seq data with DESeq2. Genome Biol. 2014;15: 550 10.1186/s13059-014-0550-8 25516281 PMC4302049

[90] Bray NL, Pimentel H, Melsted P, Pachter L. Near-optimal probabilistic RNA-seq quantification. Nat Biotechnol. 2016;34: 525–527. 10.1038/nbt.3519 27043002

[91] Anders S, Pyl PT, Huber W. HTSeq—a Python framework to work with high-throughput sequencing data. Bioinformatics. 2015;31: 166–169. 10.1093/bioinformatics/btu638 25260700 PMC4287950

[92] Langmead B, Salzberg SL. Fast gapped-read alignment with Bowtie 2. Nat Methods. 2012;9: 357–359. 10.1038/nmeth.1923 22388286 PMC3322381

[93] Li B, Dewey CN. RSEM: accurate transcript quantification from RNA-Seq data with or without a reference genome. BMC Bioinformatics. 2011;12: 323 10.1186/1471-2105-12-323 21816040 PMC3163565

[94] Harrow J, Frankish A, Gonzalez JM, Tapanari E, Diekhans M, Kokocinski F, et al GENCODE: the reference human genome annotation for The ENCODE Project. Genome Res. 2012;22: 1760–1774. 10.1101/gr.135350.111 22955987 PMC3431492

[95] DeLuca DS, Levin JZ, Sivachenko A, Fennell T, Nazaire M-D, Williams C, et al RNA-SeQC: RNA-seq metrics for quality control and process optimization. Bioinformatics. 2012;28: 1530–1532. 10.1093/bioinformatics/bts196 22539670 PMC3356847

[96] Li H, Durbin R. Fast and accurate short read alignment with Burrows-Wheeler transform. Bioinformatics. 2009;25: 1754–1760. 10.1093/bioinformatics/btp324 19451168 PMC2705234

[97] Li H, Handsaker B, Wysoker A, Fennell T, Ruan J, Homer N, et al The Sequence Alignment/Map format and SAMtools. Bioinformatics. 2009;25: 2078–2079. 10.1093/bioinformatics/btp352 19505943 PMC2723002

[98] Zhang Y, Liu T, Meyer CA, Eeckhoute J, Johnson DS, Bernstein BE, et al Model-based analysis of ChIP-Seq (MACS). Genome Biol. 2008;9: R137 10.1186/gb-2008-9-9-r137 18798982 PMC2592715

[99] Machanick P, Bailey TL. MEME-ChIP: motif analysis of large DNA datasets. Bioinformatics. 2011;27: 1696–1697. 10.1093/bioinformatics/btr189 21486936 PMC3106185

[100] Ramírez F, Dündar F, Diehl S, Grüning BA, Manke T. deepTools: a flexible platform for exploring deep-sequencing data. Nucleic Acids Res. 2014;42: W187–91. 10.1093/nar/gku365 24799436 PMC4086134

[101] Thorvaldsdóttir H, Robinson JT, Mesirov JP. Integrative Genomics Viewer (IGV): high-performance genomics data visualization and exploration. Brief Bioinform. 2013;14: 178–192. 10.1093/bib/bbs017 22517427 PMC3603213

