# Supplementary figures and images for: p73 regulates epidermal wound healing and induced keratinocyte programming
Source: PLoS One. 2019 Jun 19;14(6):e0218458. doi: 10.1371/journal.pone.0218458 (PMC6583996; doi:10.1371/journal.pone.0218458)

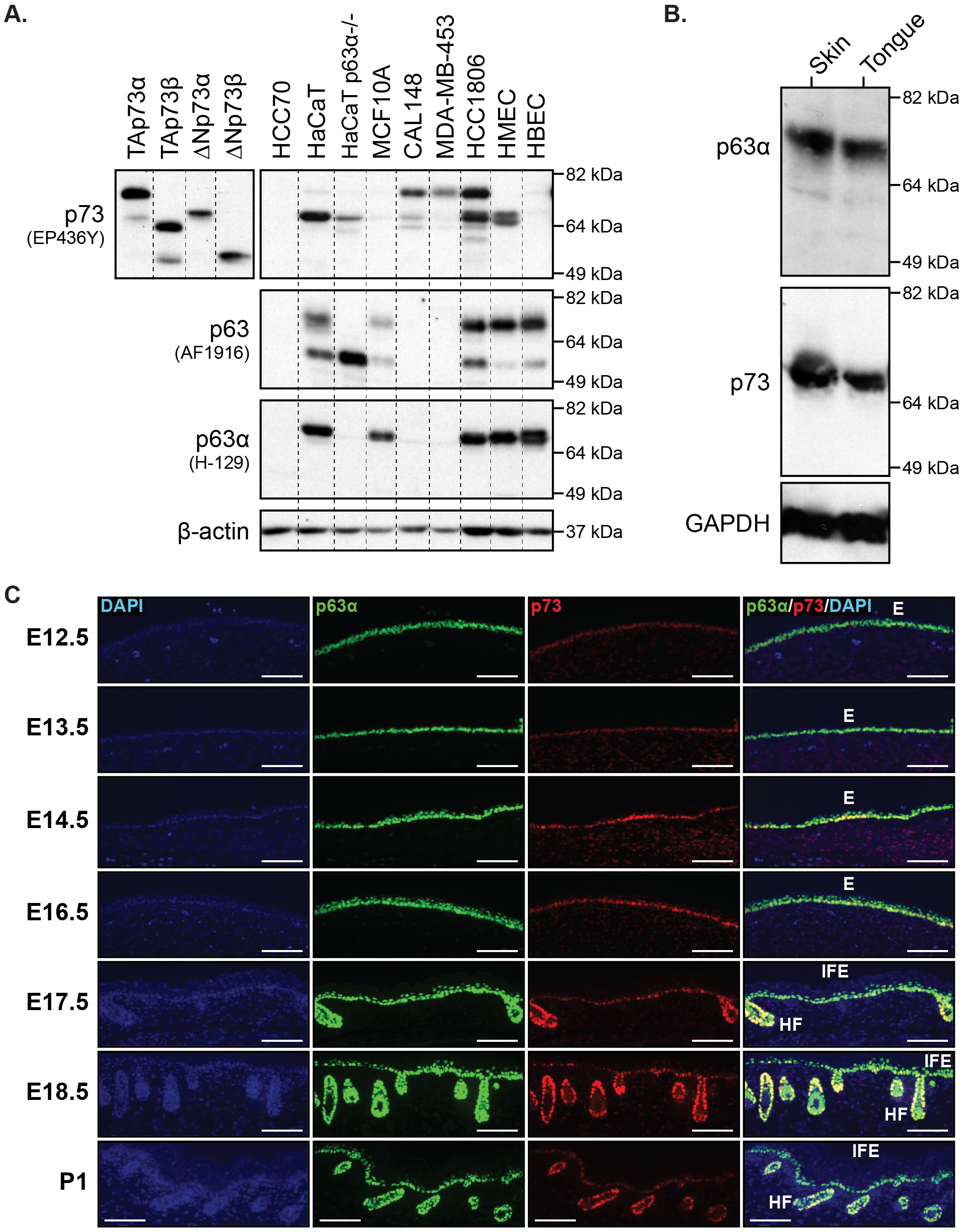

Supplement: S1 Fig — (A) Immunoblot of p73 (EP436Y), p63 (AF1916), and p63α (H-129) protein expression in a diverse set of primary and transformed human epithelial cells with varying levels of p73 and p63 mRNA expression. Human p73 isoform controls (generated by ectopic overexpression in 293FT cells) were included in the analysis to assess the sensitivity and specificity of the pan-p73 antibody. HCC70 (triple-negative breast cancer) cells lack expression of p73 and p63 mRNA and were selected to evaluate antibody specificity. HaCaT p63α-/- cells lack p63α expression as a result of CRISPR-Cas9 genomic editing. (B) Immunoblot of p63α and p73 protein expression in murine skin and tongue. Tissue was harvested from p73+/+ mice for immunoblot analysis. (C) Representative micrographs of IF staining for DAPI (blue), p63α (green), and p73 (red) in skin specimens at the indicated stages of murine development. Scale bars represent 50 μm. Regions of the skin in micrographs are labeled as: epidermis (E), IFE, and HF. (TIF) [file pone.0218458.s001.tif]

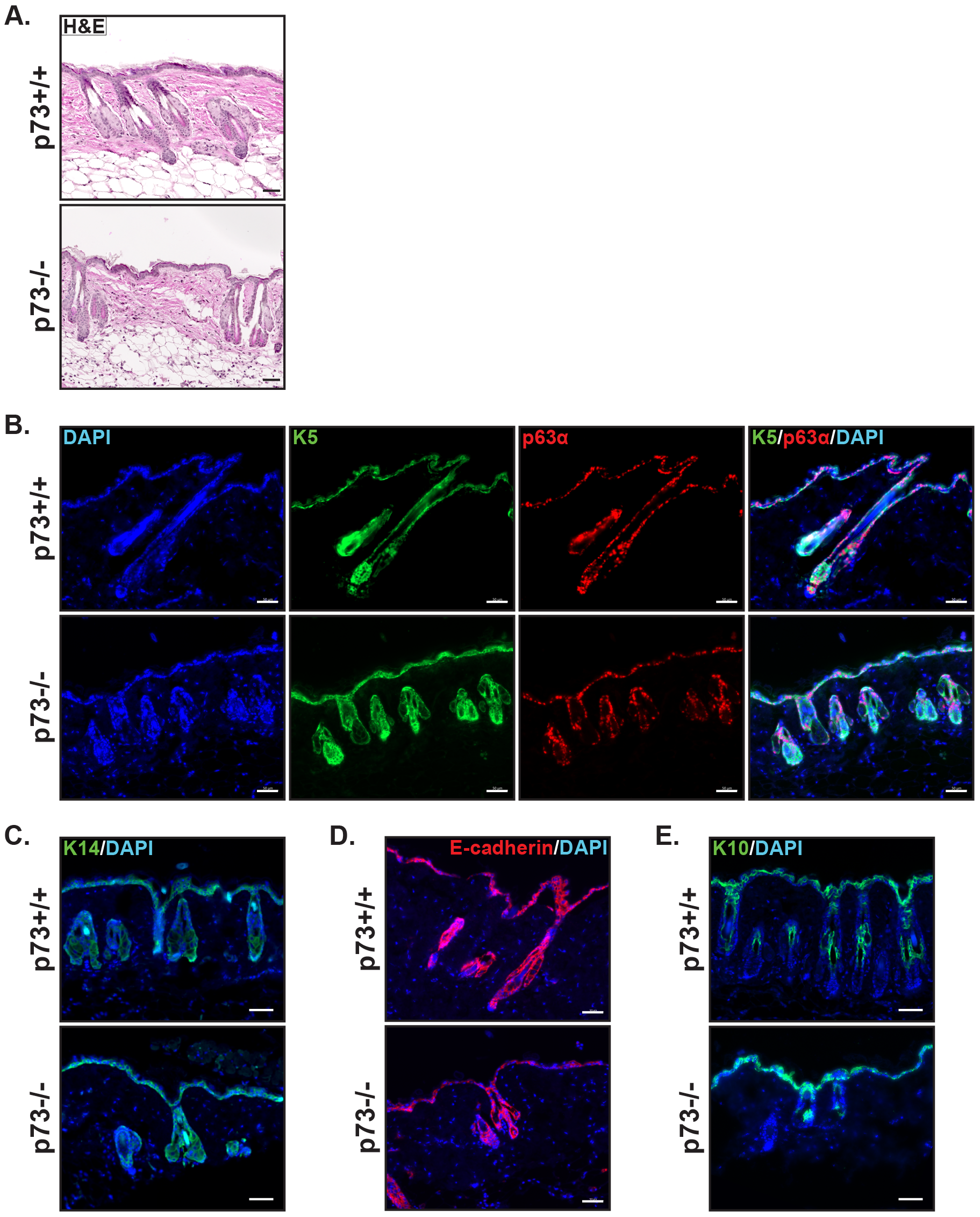

Supplement: S2 Fig — (A) Representative H&E micrographs of p73+/+ (top) and p73-/- (bottom) murine skin. (B-E) Representative micrographs of IF staining in p73+/+ (top) and p73-/- (bottom) murine skin for DAPI (blue) and: (B) K5 (green) and p63α (red), (C) K14 (green), (D) E-cadherin (red), (E) K10 (green). All scale bars represent 50 μm. (TIF) [file pone.0218458.s002.tif]

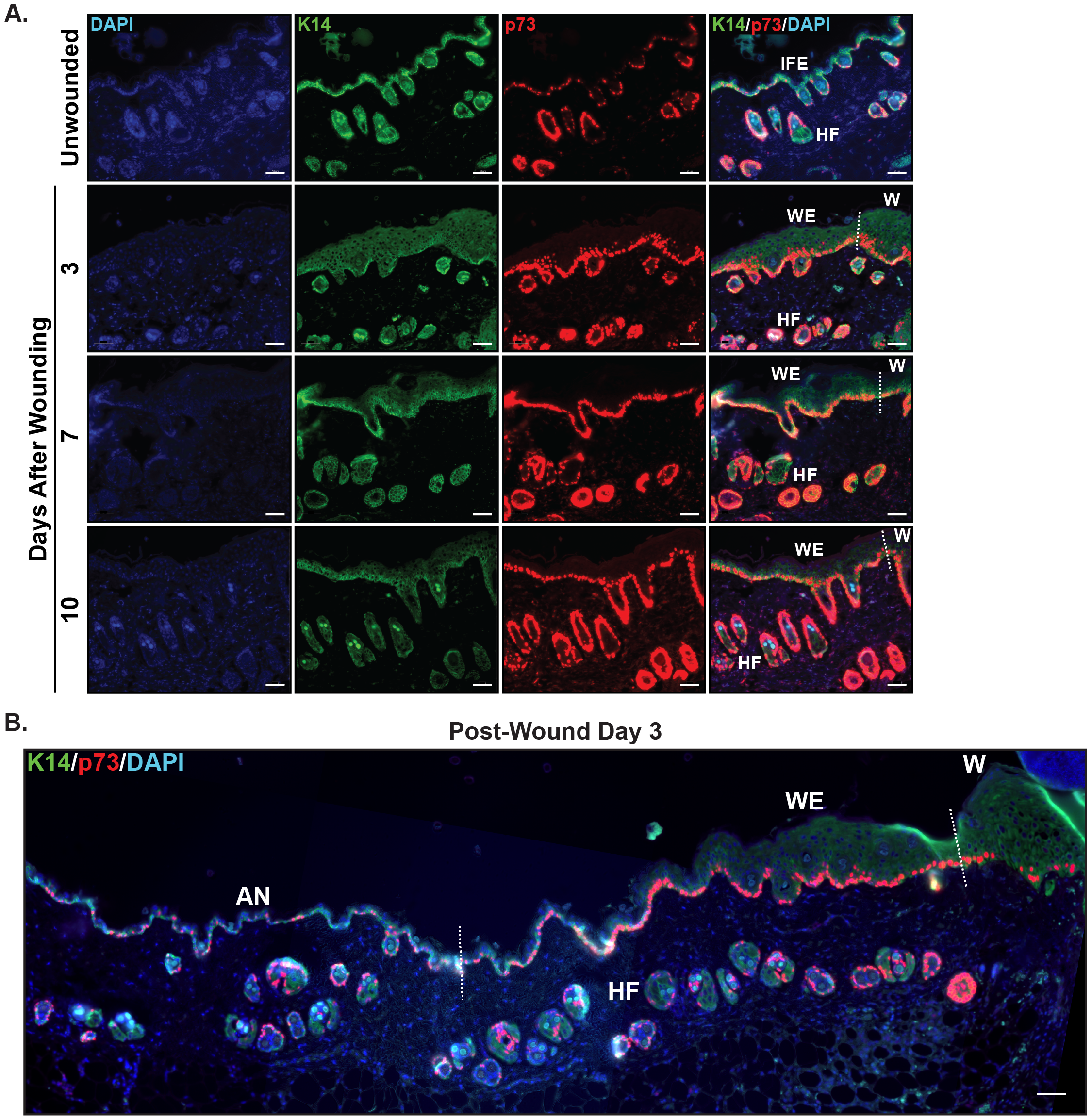

Supplement: S3 Fig — Representative micrographs of IF staining for DAPI (blue), K14 (green), and p73 (red) in skin specimens from p73+/+ mice: (A) unwounded and 3, 7, and 10 days after wounding, and (B) post-wound day 3 (zoomed out view). All scale bars represent 50 μm. Regions of the skin are labeled as: IFE, HF, epidermal wound edge (WE), newly-formed wound epidermis (W), and adjacent normal epidermis (AN). The white dotted lines indicate the border between the WE and W or AN. (TIF) [file pone.0218458.s003.tif]

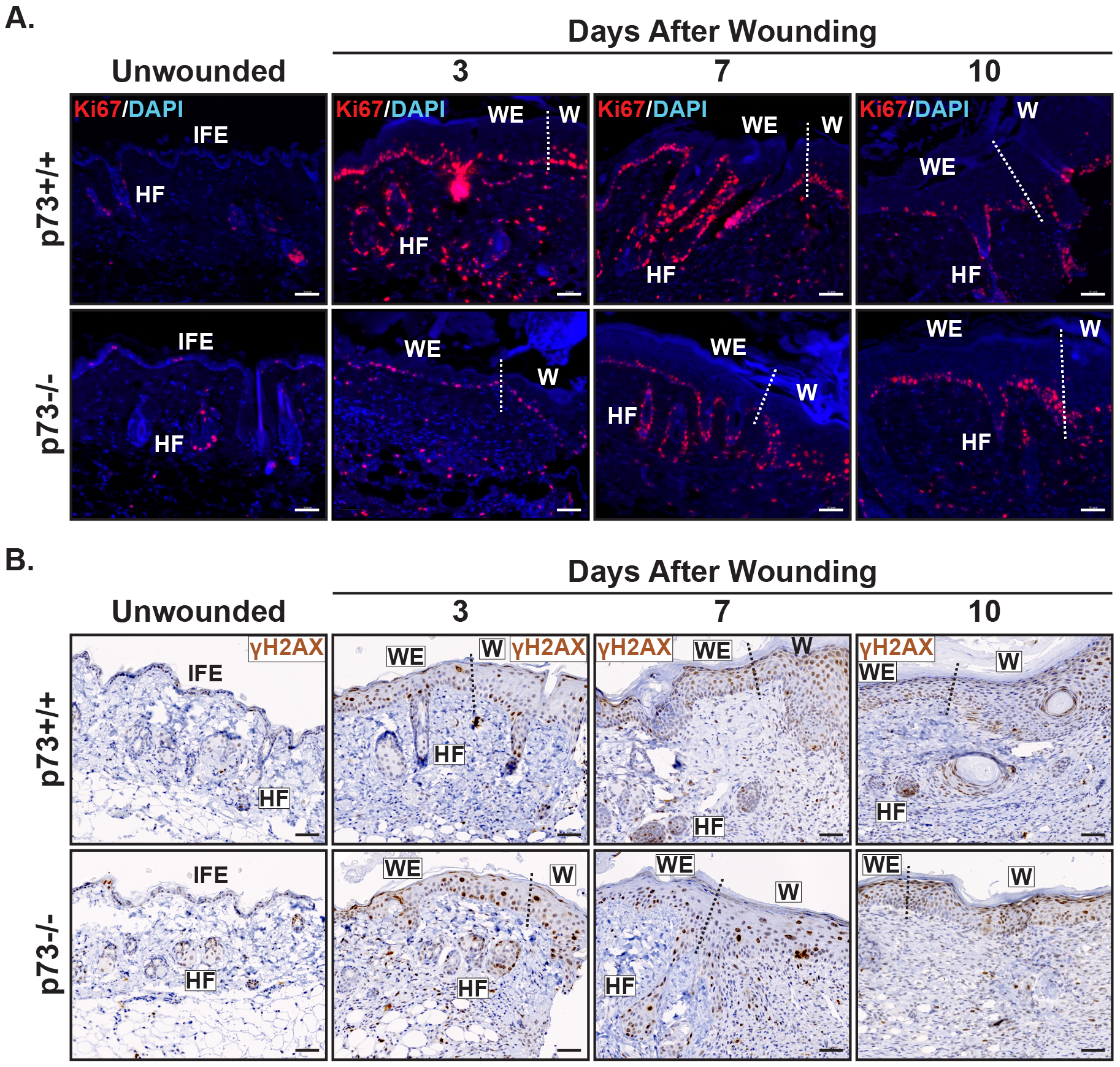

Supplement: S4 Fig — (A) Representative micrographs of IF staining for DAPI (blue) and Ki67 (red) in unwounded and wounded (days 3, 7, and 10) skin specimens from p73+/+ and p73-/- mice. (B) Representative micrographs of immunohistochemistry (IHC) staining for γH2AX in unwounded and wounded (days 3, 7, and 10) skin specimens from p73+/+ and p73-/- mice. All scale bars represent 50 μm. Regions of the skin are labeled as: IFE, HF, epidermal wound edge (WE), and newly-formed wound epidermis (W). The dotted lines indicate the border between the WE and W. (TIF) [file pone.0218458.s004.tif]

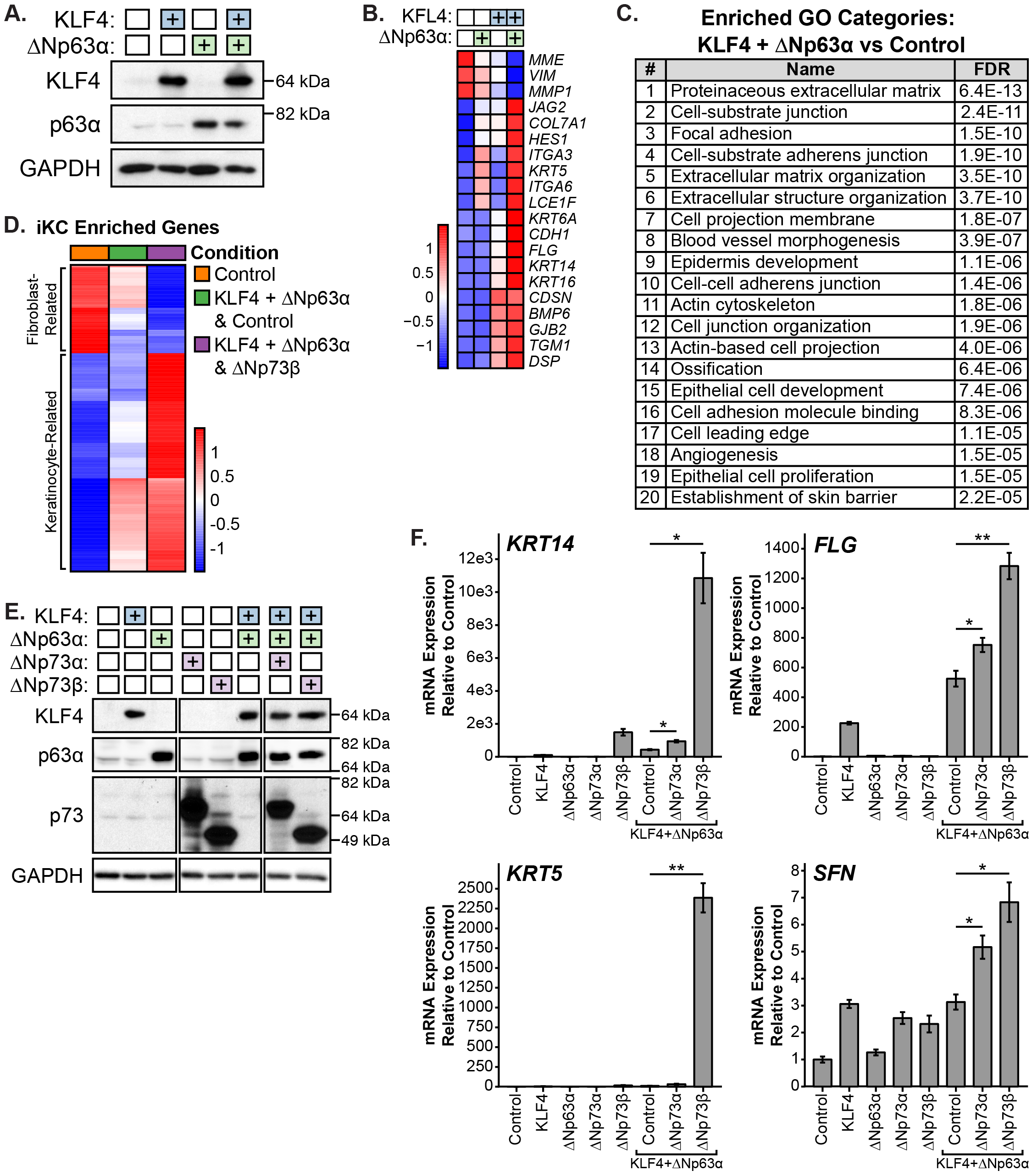

Supplement: S5 Fig — (A) Immunoblot of KLF4 and p63α protein expression in HDFn cells infected with lentivirus encoding KLF4, ΔNp63α, KLF4 + ΔNp63α, or empty vector control. Cells were grown for 3 days and protein was harvested for immunoblot analysis. (B) Heatmap of the expression of iKC-related genes in HDFn cells infected with lentivirus encoding KLF4, ΔNp63α, KLF4 + ΔNp63α, or empty vector control. Cells were grown for 3 days and RNA was harvested for RNA-seq analysis. (C) Table listing the top 20 enriched GO categories for genes differentially expressed between KLF4 + ΔNp63α and empty vector control infections from (B). (D) Heatmap of the expression of the top 250 genes contributing to PC1 from Fig 4C. HDFn cells were infected with lentivirus encoding empty vector control, KLF4 + ΔNp63α & control, or KLF4 + ΔNp63α & ΔNp73β; grown for 6 days, and RNA was harvested for RNA-seq analysis. (E) Immunoblot of KLF4, p63α, and p73 protein expression in MDA-MB-231 cells infected with lentivirus encoding ΔNp73 isoforms (ΔNp73α and ΔNp73β) or empty vector control in combination with KLF4 and ΔNp63α. Cells were grown for 4 days and protein was harvested for immunoblot analysis. (F) Bar graphs of RNA expression for the indicated iKC marker genes in MDA-MB-231 cells infected in (E). Cells were grown for 4 days and RNA was harvested for qRT-PCR analysis. Expression data are represented as the fold increase relative to control. The mean of three replicates is shown with error bars representing SEM. *p-value < 0.05, **p-value < 0.01, ***p-value < 0.001. (TIF) [file pone.0218458.s005.tif]

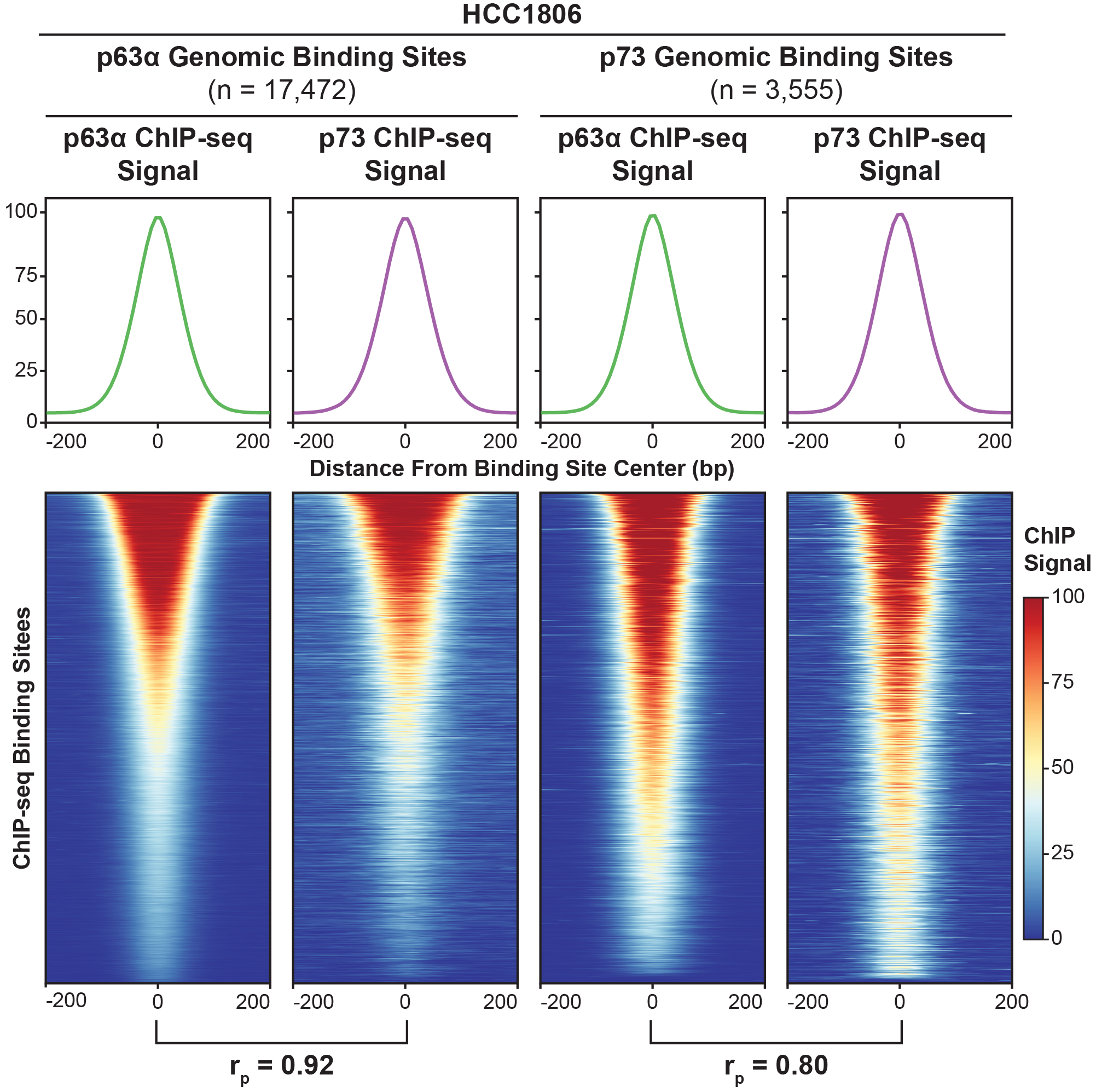

Supplement: S6 Fig — Profile plots (top panel) and heatmaps (bottom panel) of p63α and p73 ChIP-seq signal in HCC1806 cells at p63α (two leftmost panels) and p73 (two rightmost panels) genomic binding sites. Correlation between p63α and p73 ChIP-seq signal was quantified using Pearson’s correlation coefficient (rp) with deepTools [100]. (TIF) [file pone.0218458.s006.tif]
